# Zoledronate dysregulates fatty acid metabolism in renal tubular epithelial cells to induce nephrotoxicity

**DOI:** 10.1007/s00204-017-2048-0

**Published:** 2017-09-04

**Authors:** Lili Cheng, Mengmeng Ge, Zhou Lan, Zhilong Ma, Wenna Chi, Wenhua Kuang, Kun Sun, Xinbin Zhao, Ye Liu, Yaqian Feng, Yuedong Huang, Maoguo Luo, Liping Li, Bin Zhang, Xiaoyu Hu, Lina Xu, Xiaohui Liu, Yi Huo, Haiteng Deng, Jinliang Yang, Qiaoran Xi, Yonghui Zhang, Julie A. Siegenthaler, Ligong Chen

**Affiliations:** 1 School of Pharmaceutical Sciences, Tsinghua University, 100084 Beijing, China; 2 School of Life Sciences, Tsinghua University, Beijing, China; 3 Collaborative Innovation Center for Biotherapy, State Key Laboratory of Biotherapy and Cancer Center, West China Hospital, West China Medical School, Sichuan University, Chengdu, China; 4 Institute of Immunology, School of Medicine, Tsinghua University, Beijing, China; 5 Technology Center for Protein Sciences, School of Life Sciences, Tsinghua University, Beijing, China; 6 MOE Key Laboratory of Bioinformatics, School of Life Sciences, Tsinghua University, Beijing, China; 7 Department of Pediatrics, Denver-Anschutz Medical Campus, University of Colorado, Aurora, USA

**Keywords:** Zoledronate, TGFβ1 signaling, Fatty acid transporter, Lipid accumulation, Renal fibrosis

## Abstract

Zoledronate is a bisphosphonate that is widely used in the treatment of metabolic bone diseases. However, zoledronate induces significant nephrotoxicity associated with acute tubular necrosis and renal fibrosis when administered intravenously. There is speculation that zoledronate-induced nephrotoxicity may result from its pharmacological activity as an inhibitor of the mevalonate pathway but the molecular mechanisms are not fully understood. In this report, human proximal tubular HK-2 cells and mouse models were combined to dissect the molecular pathways underlying nephropathy caused by zoledronate treatments. Metabolomic and proteomic assays revealed that multiple cellular processes were significantly disrupted, including the TGFβ pathway, fatty acid metabolism and small GTPase signaling in zoledronate-treated HK-2 cells (50 μM) as compared with those in controls. Zoledronate treatments in cells (50 μM) and mice (3 mg/kg) increased TGFβ/Smad3 pathway activation to induce fibrosis and kidney injury, and specifically elevated lipid accumulation and expression of fibrotic proteins. Conversely, fatty acid transport protein Slc27a2 deficiency or co-administration of PPARA agonist fenofibrate (20 mg/kg) prevented zoledronate-induced lipid accumulation and kidney fibrosis in mice, indicating that over-expression of fatty acid transporter SLC27A2 and defective fatty acid β-oxidation following zoledronate treatments were significant factors contributing to its nephrotoxicity. These pharmacological and genetic studies provide an important mechanistic insight into zoledronate-associated kidney toxicity that will aid in development of therapeutic prevention and treatment options for this nephropathy.

**Electronic supplementary material:**

The online version of this article (doi:10.1007/s00204-017-2048-0) contains supplementary material, which is available to authorized users.

## Introduction

Zoledronate is a highly potent nitrogen-containing bisphosphonate that is commonly used to treat osteoporosis, hypercalcemia of malignancy and osteolytic bone metastases (Munier et al. 2005; Perazella and Markowitz 2008; Roelofs et al. 2006). It inhibits farnesyl diphosphate synthase (FPPS), an enzyme in the mevalonate pathway for cholesterol biosynthesis, and this inhibition reduces post-translational lipid modification (prenylation) of small GTPases (Roelofs et al. 2006). Unbound zoledronate is excreted unmetabolized through the kidney (Markowitz et al. 2003; Munier et al. 2005). Despite the clinical importance of zoledronate in several therapeutic areas, numerous cases of zoledronate-associated nephrotoxicity have been reported, which are shown to lead to renal failure, acute tubular necrosis and renal fibrosis characterized by tubular cell degeneration, loss of brush border, and apoptosis, when given intravenously (Chang et al. 2003; Markowitz et al. 2003; McKay et al. 2014; Munier et al. 2005; Ott 2012; Papapetrou 2009; Perazella and Markowitz 2008; Verhulst et al. 2015). The mechanisms of zoledronate-induced kidney toxicity are thought to be similar to its pharmacological effects in osteoclasts. The inhibition of FPPS by zoledronate may cause reduced levels of prenylated proteins leading to reduced function of Na^+^-K^+^-ATPase, aberrant integrin signaling, impaired endosomal trafficking and, ultimately, apoptosis (Luhe et al. 2008; Ott 2012; Papapetrou 2009; Perazella and Markowitz 2008). Acute tubular necrosis and fibrosis are the primary pathologies observed in zoledronate-induced renal injury, with toxicity directed at the tubular epithelium (Bergner et al. 2015; Markowitz et al. 2003; Jennings et al. 2015). However, the mechanism underlying zoledronate-induced renal toxicity remains unclear.

There is increasing evidence of a key role of disorders of fatty acid (FA) metabolism in the mechanism of renal disease and injury (Herman-Edelstein et al. 2014; Kang et al. 2015; Susztak et al. 2005). FA β-oxidation (FAO) is the major source of energy for healthy tubular epithelial cells (Kang et al. 2015), but excessive FA loads in kidney tissues result in toxicity in the proximal tubular epithelial cells and contribute to the development of tissue fibrosis and kidney disease (Kang et al. 2015; Susztak et al. 2005). TGFβ signaling, the main pathway in fibrosis development and acute renal failure (Bottinger and Bitzer 2002), plays a key role in pathogenic FAO in the kidney (Kang et al. 2015). It inhibits FAO to induce renal lipid accumulation by decreasing expression of PPAR and its signaling component *PPARγ*-*coactivator 1 alpha* (*PPARGC1A)*, the critical transcription factors required for expression of FAO pathway enzymes (Kang et al. 2015).

In this study, in vitro and in vivo models were employed to dissect the molecular mechanism of zoledronate-induced renal toxicity. Following zoledronate treatment of normal human kidney epithelial cells HK-2 cells, we used proteomics and metabolomics to systematically investigate the phenotypes and molecular pathways. We identified significant disruptions in the TGFβ pathway, FA metabolism and small GTPase signaling following zoledronate treatment of HK-2 cells. Using mouse models of zoledronate-induced renal toxicity, we showed that increased FA accumulation and fibrosis in zoledronate-treated animals were due to a TGFβ-mediated increase in renal FA transporter SLC27A2 and defective FAO. Collectively, our analyses provide compelling evidence that zoledronate-induced kidney fibrosis and tubulopathy are caused by over activation of TGFβ signaling pathways.

## Materials and methods

### Reagents

Zoledronate and fenofibrate were purchased from Tokyo Chemical Industry (Tokyo, Japan); Human TGFβ1 was purchased from PeproTech (Rocky Hill, USA) and SB431542 was obtained from Harveybio (Beijing, China); 3-(4,5-dimethylthiazol-2-yl)-2, 5-diphenyltetrazolium bromide (MTT) was purchased from Sigma-Aldrich Biotechnology (St. Louis, MO, USA); Annexin V-FITC Apoptosis Detection Kit was obtained from KeyGEN Biotech. Co., Ltd (Nanjing, China); Dulbecco’s Modified Eagle’s Medium (DMEM), Fetal bovine serum (FBS), penicillin–streptomycin, 0.25% trypsin and phosphate buffer saline (PBS) were purchased from Gibco (Grand Island, NY, USA); Saline, hematoxylin and eosin (H&E), periodic acid schiff (PAS) and Masson’s trichrome were purchased from Solarbio (Beijing, China), the Oil Red O (ORO) solution (0.5% in isopropanol) was purchased from Sigma-Aldrich Biotechnology; Human TGFβ1 ELISA kit was purchased from BOSTER (Wuhan, China). The antibodies as follows: TGFβ (CST-3709), p-SMAD3 (CST-9520), SMAD3 (BOSTER BM3559), Fibronectin (FN1) (BOSTER BA1771), Collagen I (BOSTER BA0325), α-smooth muscle actin (α-SMA) (CST-19245), ROCK (Abcam ab45171), Ras (Abcam ab52939), Cofilin (Abcam ab134963), NOX4 (BBI D121050), SLC27A2 (Proteintech 14048-1-AP), CD36 (CST-14347), Rho-GTPase antibody sampler kit (CST-9968), Combo Rho A/Rac1/Cdc42 Activation Assay Kit (Cytoskeleton BK030), SLC2A1 (CST-12939), PDHB (CST-3205), CPT1A (Proteintech 15184-1-AP), PPARA (BOSTER BA1691), β-Tubulin (EASYBIO BE0025), Na^+^-K^+^-ATPase (Abcam ab76020).

### Cell cultures

HK-2 (CRL-2190) and HEK 293T (CRL-3216) were purchased from ATCC (Manassas, VA, USA); NRK-52E was given as a present by Prof. Baoxue Yang (Peking University). All the cells were cultured in DMEM supplemented with 10% FBS and 100 IU/ml penicillin–streptomycin at 37 °C in a humid atmosphere with 5% CO_2_.

### Cell viability assay

Cell viability was measured by using MTT assay. Briefly, HK-2 cells were seeded into a 96-well plate and exposed to zoledronate (0, 0.1, 1, 5, 10, 50 µM). After treatment for varying periods of time (24, 36, 48, 60, or 72 h), 20 μl MTT at 5 mg/ml was added to each well. The cells were incubated at 37 °C for another 4 h and then dimethyl sulfoxide (DMSO) (Thermo Fisher Scientific, USA) was added to each well. The absorbance was detected at 570 nm with a microplate reader (Molecular Devices SpectraMax M5, PerkinElmer, USA). Cell viability was expressed as a percentage of the control culture.

### Flow cytometry with Annexin V/PI staining

The HK-2 cells were treated with zoledronate (0, 1, 10, 50 µM) for 48 h. Apoptosis rates were determined by flow cytometry (BD Biosciences, San Jose, CA, USA). Briefly, cells were washed with PBS once and collected by centrifuging at 1,200×*g* for 5 min, and then incubated with Annexin V-FITC (dilution 1:50) and PI (dilution 1:50) (KeyGEN Biotech) in binding buffer for 15 min in the dark at room temperature. Double-stained cells were analyzed immediately using flow cytometry and identified as apoptotic cells.

### Proteomics of HK-2 cells with and without zoledronate treatment

After treatment with zoledronate (0 and 50 µM) for 48 h, cells were lysed using urea lysis buffer (Solarbio), and then were scraped and transferred to a 1.5 ml tube, incubated at 4 °C for 30 min, centrifuged for 10 min and protein concentrations were measured using the Bradford method. 200 mg of proteins from sample were reduced with 1 mM dithiothreitol (DTT) and alkylated with 5.5 mM iodoacetamide. Proteins were digested with trypsin for overnight, and stopped by 10% trifluoroacetic acid. The peptides were desalted using C_18_ Sep-Pak cartridges and eluted with 1 ml methanol. After centrifugation, peptides were redissolved in tetraethylammonium bromide and labeled using TMT sixplex labeling reagent. The TMT-labeled peptides were combined and desalted by C_18_ Sep-Pak cartridges. The fractions were centrifuged and analyzed by LC–MS/MS. The instruments and the data analysis methods were the same as described previously (Zhao et al. 2017).

### Metabolomics of HK-2 cells with and without zoledronate treatment

After treatment with zoledronate (0 and 50 µM) for 48 h, HK-2 cells were washed three times by PBS, and then 1 ml of 80% methanol was added and incubated at −80 °C for 3 h. Cells were harvested and isolated by centrifugation at 14,000×*g* for 20 min at 4 °C. The protein concentration of the pellet was measured by the BCA assay kit (Solarbio) for normalization. The metabolite-containing supernatant of cells was transferred to a new tube and dried under nitrogen flow. The dried samples were stored in −80 °C freezer for subsequent analysis. The instruments and the data analysis methods were the same as described previously (Zhao et al. 2017).

### Treatment of zoledronate or TGFβ1 and TGFβ receptor I inhibitor SB431542

HK-2 cells were seeded into 60 mm dishes and exposed to zoledronate (0, 50 µM) or human TGFβ1 (1 ng/ml) at 37 °C for 48 h, and then SB431542 (10 µM) treatment or DMSO control was added to the cell culture supernatant and harvested 1 h later for analysis.

### Examination of ultrastructural changes by transmission electron microscopy (TEM)

HK-2 cells were treated 48 h with zoledronate and then chemically fixed with 2.5% glutaraldehyde buffered in 0.1 M PBS, pH 7.2. Cells were harvested with a cell scraper, washed with 0.1 M PBS, pH 7.2, and embedded in 2% agarose. Staining was performed with 1% osmium tetroxide for 50 min and with 1% uranyl acetate/1% phosphotungstic acid for 1 h. Dehydration of samples was done using graded acetone series. Specimens were embedded in Spurr epoxy resin and incubated for polymerization at 65 °C for 24 h. Sections were inspected with a TEM (H-7650, Hitachi, Japan).

### BODIPY staining

Lipid droplets were stained with BODIPY 558/568 C_12_ (Thermo Fisher). After HK-2 cells were treated for 48 h by zoledronate, they were incubated with the dye at a final concentration of 20 μg/ml in PBS for 45 min at 37 °C, and rinsed to remove excess stain followed by a further incubation of 1 h at 37 °C in cell culture medium. Cells were fixed with 4% paraformaldehyde at 4 °C for 30 min. Fixed cells were washed with PBS before mounting on slides using Citifluor antifadent solutions (Citifluor Ltd, United Kingdom). Samples were analyzed using a BX63 fluorescence microscope.

### Triglyceride (TG) measurement

All samples were measured using quantification kits (Jiancheng Bioengineering, Nanjing, China) according to the manufacturer’s instructions, and then absorbance was determined by EnVision Microplate reader (Molecular Devices SpectraMax M5, PerkinElmer, USA).

### Palmitic oxidation and palmitate uptake assay

Palmitic oxidation was performed according to Chen’s protocol (Chen et al. 2014). Briefly, HK-2 cells, followed by zoledronate (0, 50 μM), were incubated in the reaction medium (the same as Chen’s protocol). Reactions performed in a sealed flask were allowed to proceed for 30 min in a shaking water bath at 37 °C; 1 ml 3 M perchloric acid was added in the reaction medium to precipitate protein and nonmetabolized palmitate, and then incubated at room temperature for 2 h for collection of ^14^CO_2_ into a suspended well containing 500 μl of ethanolamine. Radioactivity was counted by a liquid scintillation counter (MicroBeta2, Perkin Elmer).

Palmitate uptake assay was similar to the aforementioned palmitate oxidation assay with the following changes (Pillon et al. 2015). HK-2 cells were cultured with [^14^C]-palmitate-containing medium for 1 h, washed three times with PBS, and then were trypsinized and transferred into glass tubes containing 3 ml methanol-chloroform (2:1), and the lipids were extracted by adding 1 ml of chloroform and 1 ml of NaCl (1 M). The lower layer was transferred into a glass tube, dried with nitrogen and redissolved with chloroform. Radioactivity was quantified by a liquid scintillation counter.

### Intracellular ROS accumulation detection

The ROS production in different samples was determined by 2,7-dichlorodihydrofluorescein diacetate (DCFH-DA) (YEASEN, Shanghai, China). Briefly, HK-2 cells were seeded in the 6-well plates at 2 × 10^5^ cells/well and pretreated with 0, 1, 10 and 50 µM zoledronate at 37 °C for 6 h, and then 500 µl DCFH-DA (10 µM final concentration) was added to each well incubated at 37 °C for 30 min. After washing three times with PBS, cells were harvested by cell scrapers and resuspended in PBS, were analyzed immediately using flow cytometry (Ex = 488 nm, Em = 525 nm).

### Membrane and cytoplasmic fractions, Rho and Rac activation assays

For extracting membrane and cytosol proteins from the cell lysate Mem-PER plus membrane protein Extraction reagent kit was purchased from Thermo Fisher Scientific (89842). RhoA and Rac1 activation were assessed using commercially available kits from Cytoskeleton (Denver, CO USA), according to the manufacturer’s instructions, with modifications. HK-2 cells were harvested using provided lysis buffer containing protease inhibitors. To detect active RhoA, equal volumes of clarified lysates were incubated with Rho-GTP binding domain of the Rhotekin protein bound to GST beads. To detect active Rac1, lysates were incubated with Rac/Cdc42 binding domain of p21-activated kinase protein bound to GST beads for 1 h at 4 °C. Active GTP-Rho, GTP-Rac, and whole cell lysates were subjected to electrophoresis on 12% acrylamide gels and analyzed via western blot techniques.

### F-actin staining

To determine the effect of zoledronate on cell cytoskeletal organization, we utilized the phalloidin immunofluorescence staining method. HK-2 cells were cultured on glass coverslips. After washing with PBS, cells were fixed with 4% paraformaldehyde for 10 min and permeabilized with 0.5% Triton X-100 in PBS for 5 min. Then the samples were incubated with FITC-phalloidin (40735ES75) in PBS for 30 min. Subsequently, the samples were counterstained with DAPI for 10 min. The images of samples coverslips were examined with BX63 fluorescence microscopy (Olympus, Japan).

### Generation of *Slc27a2*^−*/*−^ mice

The *Slc27a2* ^−*/*−^ mice model was generated using the CRISPR/Cas9 technology and was maintained on the C57BL/6J background. The candidate chimeric sgRNA targeting exon1 of *Slc27a2* (Gene ID: 26458) was designed based on http://crispr.mit.edu/. The sequence of the 20 nucleotide guide RNA used was GCGGGCGCTGCACGATCAACTGG (Underlined part is the PAM motif), and a pair of oligonucleotides for the target sequence was: forward primer 5′-TAGGCGGGCGCTGCACGATCAAC-3′ and reverse primer 5′-AAACGTTGATCGTGCAGCGCCCG-3′. In order to obtain gRNA and hCas9 mRNA, in vitro transcription reactions were respectively performed using the MEGAshortscript kit (Ambion, Am1354, USA) and mMESSAGE mMACHINE^®^ T7 Ultra Kit (Ambion, Am1345) according to the manufacturer’s instructions (Fu et al. 2016). And then, both the sgRNA and the Cas9 mRNA were purified using the MEGAclear kit (Ambion, AM1908) according to the manufacturer’s instructions. The sgRNA (30 ng/μl) was mixed with Cas9 mRNA (60 ng/μl), and the mixture was microinjected into the cytoplasm of the one-cell stage embryos. The embryos injected with RNAs were cultured in the M16 medium until the blastocyst stage, and then, approximately 15–25 blastocysts were implanted in pseudo-pregnant female mice. Genomic DNA was extracted from the tail tips of the newborn pups digested by proteinase K. The genomic sequences around the gRNA target sites were PCR amplified using the following primers: forward primer 5′-CTCCAAGATGTGCGGTACTT-3′ and reverse primer 5′-GCAGAGACTTGGCACGAATG-3′. The PCR products were purified using TIANquick Midi Purification Kit (Tiangen, Beijing, China) and sequenced directly. Over the ten generations of breeding, *Slc27a2* ^−*/*−^ mice can be used for experiments.

### Animal experiments

The wild type C57BL/6 J mice (8 weeks old) were supplied by laboratory animal research center of Tsinghua University (Beijing, China). The male wild type and *Slc27a2* ^−*/*−^ mice were housed in an animal care facility at Tsinghua University, under controlled conditions of temperature (22 ± 2 °C) and relative humidity (50 ± 20%) with a 12 h light/dark cycle with water and food freely available. The mice were randomly allocated to the vehicle (0.9% saline)–treated (control, *n* = 5), zoledronate (3 mg/kg/week)–treated (Zole, *n* = 5) and zoledronate (3 mg/kg/week)/fenofibrate (20 mg/kg) co-treated (Zole + fenofibrate, *n* = 5) groups. And the *Slc27a2* ^−*/*−^ mice were randomly allocated to the vehicle (0.9% saline)–treated (control, *n* = 5) and zoledronate (3 mg/kg/week)–treated (Zole, *n* = 5) groups. 1 day before the zoledronate injection, the Pparα agonist fenofibrate (20 mg/kg for 3 days) was administered by oral gavage, and all animals were injected zoledronate via the tail vein each week and treated for 4 weeks.

Following final treatment and collection of urine, kidney tissues were harvested and cut in cross section. Part was fixed in 4% paraformaldehyde for morphological examination and the others kidney was snap frozen in liquid nitrogen and then stored at −80 °C. All procedures performed in studies involving animals were approved by the Institutional Animal Care and Use Committee of Tsinghua University. All animal experiments were repeated three times independently.

### Biochemical markers of urine

After 2 or 4 weeks treatment, mice were maintained under fasting condition for 12 h and urine was collected using a metabolic cage that was placed over an ice bath to avoid degradation of metabolites. Urine samples were immediately centrifuged at 10,000×*g* for 10 min at 4 °C. And then the supernatant were immediately stored at −80 °C for subsequent analysis. For clinical chemistry measurements, the supernatant were analyzed for creatinine using a fully automatic biochemical analyzer (Toshiba, Tokyo, Japan).

### Determination of TGFβ1 concentration in culture supernatant and serum

The TGFβ1 concentration of HK-2 cells culture supernatant or mice serum was measured by specific enzyme-linked immunosorbent assay (ELISA) kit according to the manufacturer’s instructions. This assay has <1% cross-reactivity for TGFβ2, TGFβ3 and TGFβ5.

### RNA extraction and qPCR analysis

Total RNAs were prepared from cells or kidney tissues by using ultra-pure TRIzol reagent (Tiangen) according to the manufacturer’s instructions. Reverse transcription was performed on equal amounts of total RNA (2.5 μg) by using random hexanucleotide primers to produce a cDNA library for each sample. qPCR were run in the ABI ViiA™7 Real-Time System (Life Technologies) by using SYBR Green Master Mix (Transgen, Beijing, China) and gene-specific primers (Table S1 online). Each sample was run in triplicate, and the comparative threshold cycle (*C*t) method was used to quantify fold increase (2^−ΔΔ*Ct*^) compared with lean controls.

### Western blot

Cells or kidney tissue were washed with PBS (only cells) sonicated in RIPA lysis buffer (Beyotime, Shanghai, China) with PMSF (Beyotime), and centrifuged at 14,000×*g* for 10 min. The 2X sample loading buffer was added to the protein sample and heated at 100 °C for 10 min. The proteins were separated by electrophoresis in 10% gels, and then transferred onto PVDF membrane (Merck Millipore, Germany) with 350 mA, blocked with 5% skimmed milk for 1 h, and incubated with antibody overnight at 4 °C. Protein expression was detected using a chemiluminescent staining reagent kit to visualize the signals.

### H&E staining, ORO staining, PAS staining and Masson’s staining

Paraffin sections (5 μm) (Harada et al. 2016) stained with H&E, PAS and Masson’s trichrome, and frozen sections (6 μm) were used for ORO staining to determine the lipid accumulation. All stained sections were examined under a light microscope (Olympus,), and finally evaluated for injury by an experienced pathologist who was blinded to the treatment each animal had received.

### Immunohistochemical (IHC) staining

Kidney tissue sections (5 μm) (Harada et al. 2016) were deparaffinized in xylene, using graded ethanol, and rinsed with distilled water. For antigen retrieval, the sections were incubated with citrate buffer solution (pH: 6.0, 100 °C). After blocking by using 0.3% hydrogen peroxide in methanol for 30 min, the sections were incubated overnight at 4 °C with rabbit primary antibody Fn1, collagen I, α-SMA and Slc27a2. The secondary antibody was added and the sections were incubated at 37 °C for 1 h. An Olympus microscope was used for image analysis.

### ChIP-Seq

Smad2/3 ChIP-Seq data were downloaded from Gene Expression Omnibus (GEO: GSE53233) and aligned to mouse genome (mm10) by using Bowtie1.1.2. Both peak calling and generation of ChIP-Seq visual files were implemented by using MACS1.4.2. Gene tracks of ChIP-Seq data are the snapshots of bedgraph files visualized in IGV.

### Statistical analysis

Statistical analysis was performed using GraphPad Prism software, version 6.0. Data were expressed as mean ± standard error of mean. Unpaired *t* tests were performed for comparison between two groups. Data were analyzed using a one-way analysis of variance (ANOVA) followed by a Newman–Keuls multiple comparison test. Statistical significances were calculated and indicated.

## Results

### Proteomic profiles of HK-2 cells treated with zoledronate revealed dysregulated TGFβ signaling and FA metabolism

**Fig. 1:**
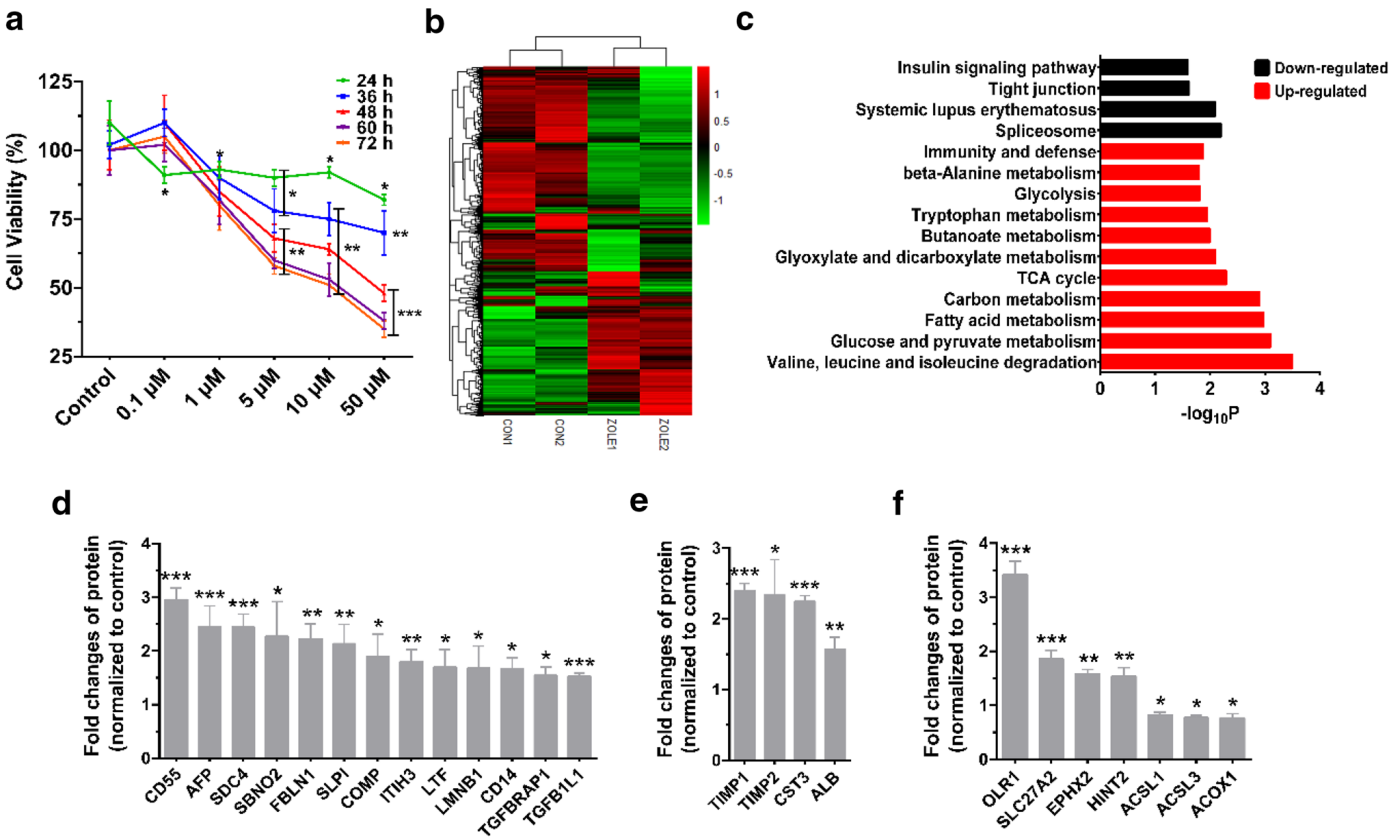
Analysis of proteomic data of HK-2 cells treated with or without zoledronate (50 µM) for 48 h. **a** Cell viability curves of HK-2 cell. HK-2 cell was treated by various doses of zoledronate (0, 0.1, 1, 5, 10, 50 µM) for 24, 36, 48, 60 and 72 h. **b** Heat map of significantly changed proteins following zoledronate treatment on HK-2 cells. **c** Gene ontology (GO) analysis of HK-2 cell treated with control and zoledronate samples. The *graph* shows the negative log *p* values for the enrichment of the specific pathways. **d** Relative protein levels related to TGFβ and inflammation. **e** Relative protein levels related to fibrosis and kidney injury. **f** Relative protein levels related to lipid and FA metabolism. Data presented as mean ± SD (each treated sample (*n* = 2) were compared with each untreated one (*n* = 2) once, resulting in four sets of data). Zole is the abbreviation of zoledronate in all the figures

To determine the cytotoxic range for zoledronate in HK-2 cells, the MTT assay showed that 50 µM zoledronate treatment for 48 h resulted in the cell viability at around 50% (Fig. 1a). Apoptotic rate, pro-apoptotic and kidney injury marker genes were significantly elevated with increasing doses of zoledronate treatments (Fig. S1a, b online). Based on the information above, 50 μM zoledronate and 48 h were used as the maximal treatment conditions to investigate the effects of zoledronate on the cytotoxicity of HK-2 cells in the subsequent experiments.

Initially, to identify the cellular and molecular pathways underlying zoledronate-induced nephrotoxicity, we applied proteomic profiling with HK-2 cells with or without 50 μM zoledronate treatment. A total of 5197 proteins were identified by LC–MS/MS, among which, 280 proteins were identified as significantly altered as defined by a corrected *p* value of 0.05 and at least 50% of up-regulation and 25% of down-regulation of their expression in 50 μM zoledronate-treated samples as compared with the untreated (Table S2 online). Down-regulated proteins were relatively less than up-regulated, so that a smaller percentage alternation was considered arbitrarily. The quantitative ratios between zoledronate and control groups are in a heat map (Fig. 1b).

GO analysis identified specific changes in lipid and FA metabolism, as well as inflammation and bioenergenesis metabolism (Figs. 1c, S2 online). Of particular interest, TGFβ and inflammation-related proteins [13 proteins such as transforming growth factor beta 1 induced transcript 1 (TGFβ1I1), transforming growth factor beta receptor associated protein 1 (TGFβRAP1), CD55, alpha fetoprotein (AFP), syndecan 4 (SDC4), strawberry notch homolog 2 (SBNO2) et al.] (Mizejewski 2015; Kim et al. 2004; van Beek et al. 2005; Karkampouna et al. 2016; González-Guerrero et al. 2017) were up-regulated with a mean ratio >1.5 (Fig. 1d). TGFβ and inflammation play critical roles in fibrosis development and acute kidney injury (Bottinger and Bitzer 2002). Consistent with elevated TGFβ-related proteins, the proteins associated with fibrosis and kidney injury (Kang et al. 2015; Sanchez-Nino et al. 2015) [TIMP metallopeptidase inhibitor-1 (TIMP1), -2 (TIMP2), cystatin C (CST3) and albumin (ALB)] (Kang et al. 2015; Mizejewski 2015) were increased significantly with zoledronate treatment (Fig. 1e). Proteins involved in FA and lipid transport including OLR1 (3.4-fold) and SLC27A2 (1.9-fold) were significantly elevated whereas proteins involved in FAO were decreased compared with those in controls (Fig. 1f). These analyses suggest that the zoledronate treatment may dysregulate TGFβ signaling and FA metabolism.

### Zoledronate activated the TGFβ/Smad cascade to induce fibrotic signaling

**Fig. 2:**
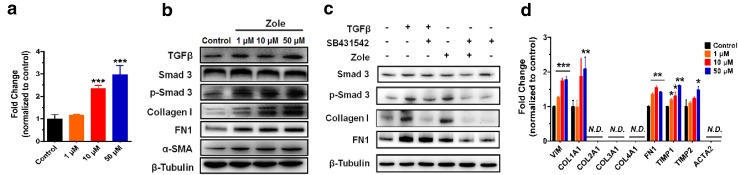
Effects of zoledronate treatments on TGFβ1 in HK-2 cells. **a** TGFβ1 mRNA expression in HK-2 cells under various concentrations of zoledronate treatments. **b** Western blot analysis of TGFβ1/SMAD3 signaling and fibrosis markers in the HK-2 cells after zoledronate treatments. **c** Comparisons of zoledronate treatment with TGFβ1 receptor agonist (TGFβ) or inhibitor (SB431542) on p-Smad3 and fibrotic factor protein expressions. **d** Induction of relative mRNA levels of genes related to kidney fibrosis by zoledronate treatments. All data are presented as mean ± SD (*n* = 6) and ^*^ *P* < 0.05, ^**^ *P* < 0.01 and ^***^ *P* < 0.001 compared to control, respectively

Proteomic data identified that the TGFβ-related signaling was up-regulated in HK-2 cells after zoledronate treatment, and TGFβ is the most powerful cytokine in kidney fibrosis development and kidney injury (Bottinger and Bitzer 2002). Quantitative real-time polymerase chain reaction (qPCR) results demonstrated that TGFβ1 expression was about 2.4-fold (10 μM) and 3.0-fold (50 μM) higher in zoledronate-treated HK-2 cells than those in the controls, indicating that TGFβ1 production was stimulated by zoledronate treatment (Fig. 2a). Therefore, TGFβ1-associated downstream genes of Smad3 and fibrotic factors were examined. Immunoblot analysis confirmed up-regulation of TGFβ1 and p-Smad3, as well as three fibrosis factors, collagen I, FN1 and α-SMA, after zoledronate treatment (Fig. 2b). Next, the effect of zoledronate, TGFβ1 and SB431542 (an inhibitor of TGFβ receptor 1), was examined on regulating the genes above. Inhibition of TGFβ receptor 1 significantly suppressed zoledronate-induced expression of p-Smad3 and fibrotic markers (Fig. 2c). On the other hand, qPCR results also confirmed that zoledronate induced over-expressions of some of key fibrosis factors such as *vimentin (VIM), collagen type I alpha 1 chain (COL1A1)* (only at 50 μM zoledronate), *FN1*, *TIMP1*, *TIMP2* (only at 50 μM zoledronate) (Fig. 2d). These results supported a TGFβ1/Smad3 cascade of signaling events triggered by zoledronate treatments for kidney fibroblast activation. In order to further validate the induction of TGFβ1 by zoledronate treatment, human embryonic kidney 293T cell line HEK 293T (Fig. S3a online) and rat renal tubular cell line NRK-52E (Fig. S3b online) were used for zoledronate treatments. TGFβ1 and down-stream targets, p-SMAD3 and collagen I, were regulated similarly to HK-2 cells, suggesting that activation of TGFβ1 signaling might be conserved in these renal cells. As a potent multifunctional cytokine, TGFβ shows both anti- and pro-inflammatory roles in different situations (Sanjabi et al. 2009). The expression of pro-inflammatory cytokine markers IL1B (3.8-fold), IL6 (6.8-fold) and TNFα (2.0-fold) were significantly increased (Fig. S3c) compared to the untreated ones, indicating that zoledronate resulted in the inflammation of the renal cells. Zoledronate-induced TGFβ over expression may play a pro-inflammatory role here. Reports have been shown that zoledronate increases the inflammatory response of kidney, macrophage and prostate cancer cells (Lin et al. 2014; Muratsu et al. 2013; Toussaint et al. 2009). These data suggest zoledronate may induce renal fibrosis and injury by triggering TGFβ/Smad3 dependent signaling pathways.

### Zoledronate caused intracellular lipid accumulation due to dysregulated FA uptake and oxidation

**Fig. 3:**
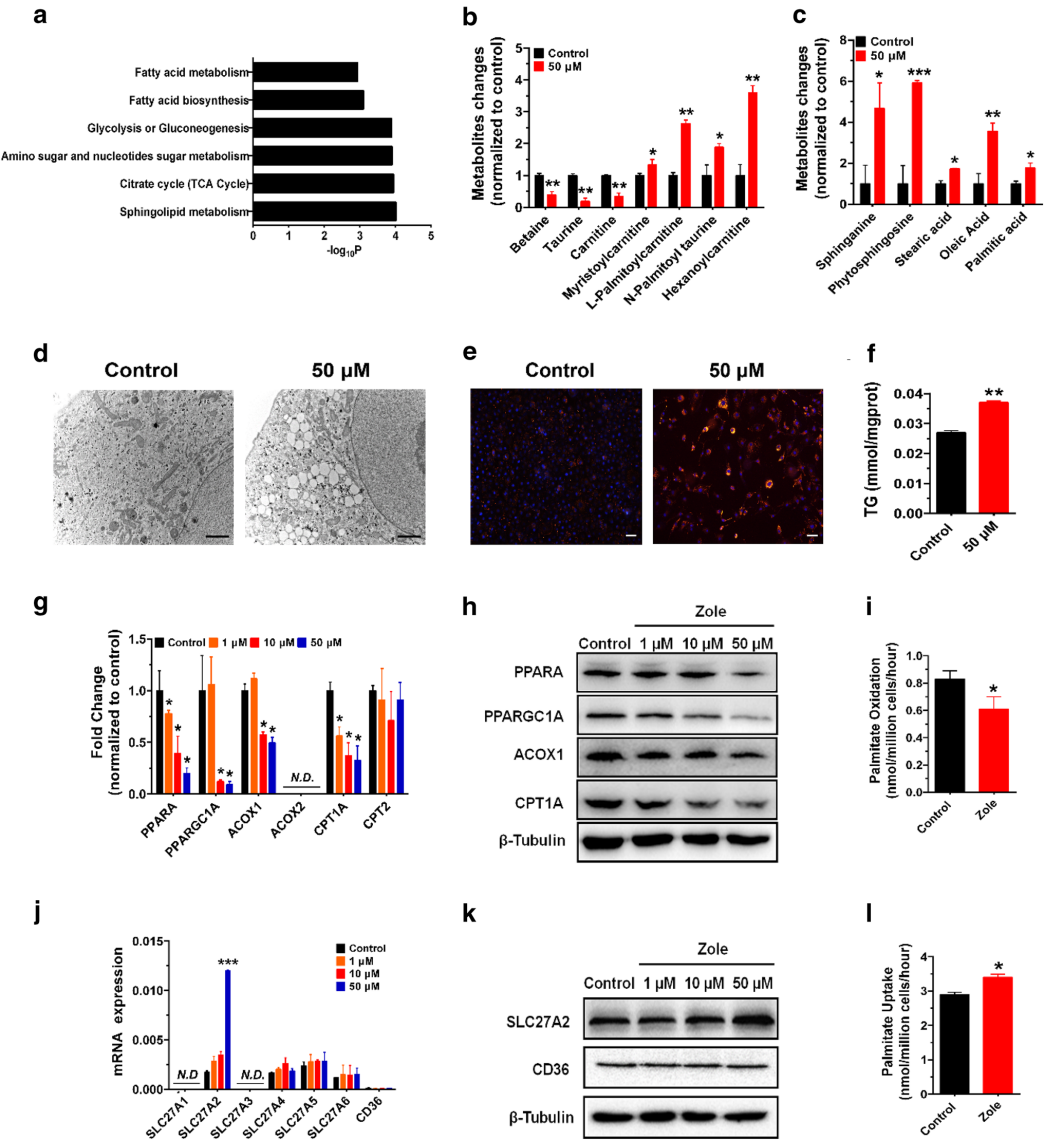
Zoledronate treatments resulting in lipid accumulation in HK-2 cells. **a** Pathway analysis of metabolomic data revealed FA metabolism as the major pathway affected in HK-2 cells treated with or without 50 µM zoledronate. **b**, **c** Elevations of metabolites of FAO carrier, conjugated FA intermediates, FAs and lipid species in 50 µM zoledronate-treated HK-2 cells as compared with the control group in metabolomic data. **d**, **e** Representative TEM (*scale bar* 2 μm) and BODIPY staining (*scale bar* 20 μm) showed lipid drop formation in 50 µM zoledronate-treated HK-2 cells. **f** TG quantifications in the samples of HK-2 cells with or without 50 µM zoledronate. **g**, **h** Relative mRNA or protein levels of genes related to FAO in the samples of HK-2 cell treated with zoledronate at doses of 0, 1, 10, 50 µM for 48 h. **i** Ability of FAO was reduced by zoledronate treatment using [^14^C]palmitate oxidation assay. **j**, **k** Relative mRNA and protein levels of genes relative to FA transport in the samples of HK-2 cell treated with zoledronate at doses of 0, 1, 10, 50 µM for 48 h. **l** Increased ability of FA uptake induced by zoledronate treatment using [^14^C]palmitate uptake assay

To further explore metabolic alternations in FA metabolism that occur with zoledronate exposure, metabolomics was used to identify metabolites that were altered in HK-2 cells following zoledronate treatment (50 μM) (Table S3 online). The top significantly altered metabolic pathways included lipid and FA metabolism, glucose metabolism and TCA cycle (Fig. 3a). Among the significantly changed metabolites, the amounts of carnitine, betaine and taurine,which are important on FAO (Fritz and Yue 1963; Ibrahim et al. 2003), were significant lower; however, FAO intermediates, such as myristoylcarnitine, l-palmitoylcarnitine, *N*-palmitoyl taurine and hexanoylcarnitine were significantly higher in the 50 μM zoledronate treatment group than those of controls (Fig. 3b). Meanwhile, 50 μM zoledronate treatment resulted in significant increase of intracellular lipids, including sphinganine, phytosphingosine, stearic acid, oleic acid and palmitic acid (Fig. 3c). These findings indicate zoledronate treatment in HK-2 cells may disrupt FA metabolism.

We next examined zoledronate’s effect on cellular lipid status using TEM and BODIPY staining for neutral lipids (Fig. 3d, e). Both analyses revealed higher cellular lipid content after zoledronate treatments, as indicated by lipid accumulation in droplets in TEM images (Fig. 3d) and elevated BODIPY labeling in zoledronate-treated cells (Fig. 3e). Further, triglyceride content was significantly increased 37.3% after 50 μM zoledronate treatment compared with controls (Fig. 3f). This, in conjunction with the metabolomics analyses, is compelling evidence that zoledronate treatment induces improper FA accumulation and may be an important component of zoledronate-induced nephrotoxicity.

In order to explore the molecular pathways related to zoledronate-induced lipid accumulation, the mRNA levels of key enzymes responsible for FAO were checked (Fig. 3g) (Kang et al. 2015). PPARA and PPARGC1A are key transcriptional regulators responsible for regulating the enzymes involved in FAO, such as a*cyl*-*CoA oxidase (ACOX1), carnitine palmitoyltransferase*-*1 alpha (CPT1A) and* -*2 (CPT2)* (Kang et al. 2015). mRNA levels of PPARA was reduced by about 22.2, 60.7 and 79.9% of that of controls at 1, 10 and 50 μM zoledronate treatments, respectively; *PPARGC1A* was reduced by about 90.0% of that of controls at both 10 μM and 50 μM zoledronate treatments (Fig. 3g). *CPT1A* was significantly reduced by about 43.8, 62.7 and 67.4% relative to the controls at 1, 10 and 50 μM zoledronate treatments, respectively; *ACOX1* was significantly decreased by about 42.5 and 50.3% relative to the controls at 10 and 50 μM zoledronate treatments, respectively (Fig. 3g). However, zoledronate did not affect the mRNA expression of *CPT2* whereas *ACOX2* was not detected (Fig. 3g). Consistent with gene expression analysis, immunoblot analysis for protein expression of revealed the deceases in PPARA, PPARGC1A, ACOX1 and CPT1A after zoledronate treatments (Fig. 3h). These results suggested that zoledronate may inhibit FAO by down-regulating *PPARA* and *PPARGC1A*, thereby suppressing *CPT1A* and *ACOX1*.

To check the FAO rate following zoledronate treatment, palmitate oxidation assay was conducted to evaluate FAO activity (Fig. 3i) (Kim et al. 2004). After 50 μM zoledronate treatment, the palmitate oxidation rate significantly decreased to 73.5% of that of controls. Next, the expression of FA transporters was investigated, followed by proteomic analysis (Fig. 1f). After 48 h zoledronate exposure, mRNA levels of *SLC27A2* were about 6.9-fold higher in 50 μM zoledronate treatment group than controls, but there were no significant changes in the mRNA levels of *CD36* and other *SLC27* family members following zoledronate treatments (Fig. 3j). Protein level of SLC27A2 was also increased at 50 μM of zoledronate treatment whereas CD36 appeared unchanged (Fig. 3k).

Increased expression of FA transporter *SLC27A2* indicates FA uptake may be increased following zoledronate treatment. To evaluate FA uptake, we applied radioisotope labeled [^14^C] palmitate uptake assay (Chen et al. 2014; Pillon et al. 2015). Palmitate uptake was significantly increased about 17.2% after zoledronate treatment compared with control treatment (Fig. 3l). *SLC27A2* plays a key role in FA uptake and biosynthesis serving as a long-chain FAs transporter that is mainly expressed in kidney and liver cells (Anderson and Stahl 2013). Collectively, our data indicate up-regulation of *SLC27A2*, along with impaired FAO, contributes to lipid accumulation following zoledronate exposure in HK-2 cells.

### Zoledronate treatment induced renal fibrotic injury and lipid accumulation in mice

**Fig. 4:**
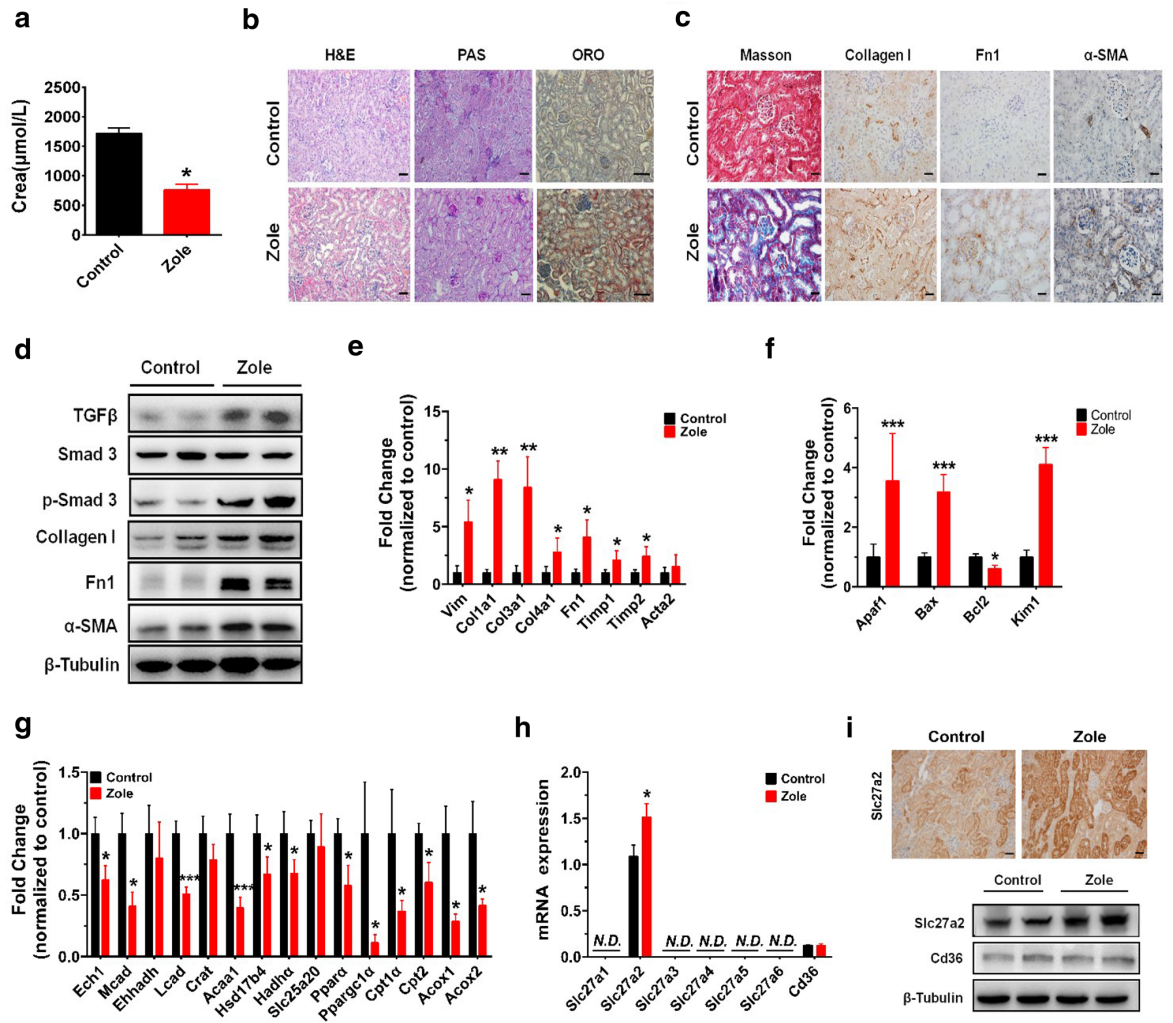
Effects of renal toxicity of zoledronate treatment in mice and its relative molecular pathways. **a** Reduced creatinine secretion in zoledronate-treated mice as compared with the control group. **b** Representative images of zoledronate untreated and treated mouse kidney sections stained with H&E, PAS and ORO staining (*scale bar* 1 mm). **c** Representative pictures of Masson’s trichrome staining (*scale bar* 50 μm) and collagen I, Fn1 and α-SMA IHC (*scale bar* 20 μm) for detection of zoledronate-induced kidney injury. **d** Western blot analysis of TGFβ1/Smad3 pathway and fibrosis markers in the kidney of controlled and zoledronate-treated mice. **e** Relative transcript levels of fibrosis and kidney-injury-related genes in controls and zoledronate-treated mice. **f** Relative mRNA levels of typical apoptosis and kidney injury factors. **g** Relative mRNA levels of FAO-related genes in controls and zoledronate-treated ones. **h** Relative transcript levels of FA uptake-related transporter or carrier in controls and zoledronate-treated ones. **i** Representative IHC images and western blot analysis of mouse kidney from control and zoledronate-treated mice for Slc27a2. (*scale bar* 20 μm). Each group had five mice and was treated for 4 weeks in the animal studies

We next examined the cellular and molecular underpinnings of zoledronate-induced renal toxicity in mice. Following 4-week treatments with zoledronate in which mice received the drug once weekly, urine creatinine was significantly decreased more than 50.0% in mice treated with zoledronate compared with vehicle treatment indicating impaired kidney function (Fig. 4a). Creatinine was not affected after 2-week treatments in mice (Fig. S4a online) thus we used the 4-week treatment regime for pathology and molecular analyses of zoledronate-induced nephrotoxicity. H&E and PAS staining revealed substantial morphological changes in renal tubules including tubular atrophy, loss of brush border and inflammatory cell infiltration (Fig. 4b). The ORO staining was elevated in zoledronate-treated animals pointing to enhance neutral lipid accumulation with zoledronate exposure (Fig. 4b). There was clear evidence of kidney fibrosis in zoledronate-treated animals, demonstrated by increased interstitial Masson’s trichrome staining, collagen I, Fn1 and α-SMA immunolabeling in the kidney cortex (Fig. 4c). These data demonstrated zoledronate-treatment in an animal model induces kidney fibrosis and FA accumulation, both of which are major contributors to kidney pathology. We next tested if pro-fibrotic pathway TGFβ1 and pro-apoptotic pathways, both identified in our HK-2 cell model, were altered in the kidney of zoledronate-treated animals. Kidney expression of TGFβ1 protein and p-Smad3 were elevated as were levels of fibrotic proteins collagen I, Fn1 and α-SMA following zoledronate treatment (Fig. 4d). To further examine the effect of zoledronate treatment on the TGFβ production, we measured the TGFβ amount in the supernatant or serum of treated or untreated HK-2 cells and animals, respectively. It showed significantly increased production of TGFβ in supernatants of zoledronate-treated cells (Fig. S4b) but not in the serum of zoledronate-treated mice (Fig. S4c), implying that zoledronate might cause TGFβ over-production in the local renal tissue but not the whole-body level. Many fibrosis genes were significantly elevated in the kidney following zoledronate treatment, including *Vim*, *Col1a1, Col3a1, Col4a1, Fn1, Timp1* and *Timp2* but not *Acta2* (Fig. 4e). mRNA levels of pro-apoptotic factor *Apaf*-*1* (3.6-fold up), *Bax* (3.2-fold up) and pro-survival factor *Bcl2* (0.4-fold down) were regulated reciprocally and *Kim*-*1* was significantly increased by more than 4.1-fold after zoledronate treatments compared to the controls, which indicated that the tubular injury induced by zoledronate caused enhanced cell apoptosis (Fig. 4f). These data point to kidney fibrosis and apoptotic cell death as part of zoledronate-induced nephrotoxicity.

We next analyzed the molecular pathways underlying zoledronate-induced lipid accumulation. FAO-relevant genes [except *enoyl*-*CoA hydratase 3*-*hydroxyacyl CoA dehydrogenase (Ehhadh), carnitine O*-*acetyltransferase (Crat) and solute carrier family 25 member 20 (Slc25a20)*] were significantly reduced in mouse renal tissue following zoledronate treatment (Fig. 4g) (Kang et al. 2015; Li et al. 2008; Polinati et al. 2009; Liu et al. 2016), pointing to defects in FAO in mouse kidney by zoledronate administration. Long-chain FAs are the main energy source for renal tubular epithelial cells (Kang et al. 2015), which enter the cells mostly through several membrane proteins, including FA carrier CD36 and FA transport proteins (Verhulst et al. 2015). Similar to our observations in HK-2 cells, *Slc27a2* was the only highly expressed FA transport protein in mouse kidney and zoledronate induced about 0.4-fold higher expression of *Slc27a2*, but did not affect *Cd36* (Fig. 4h, i). Similar to the cellular study HK-2 cells, zoledronate treatment also significantly increased the expression of pro-inflammatory cytokine markers Il1b (2.1-fold), Il6 (5.1-fold) and Tnfα (7.6-fold) as compared to the control group (Fig. S4d), which may be due to the fibrotic and lipotoxic effects of accumulation of fatty acid. These data point to disruption in FAO and transport as a major cause of lipid accumulation in zoledronate-treated animals.

### *Slc27a2* deficiency reversed the renal lipid accumulation and markedly reduced the renal toxicity induced by zoledronate treatment

**Fig. 5:**
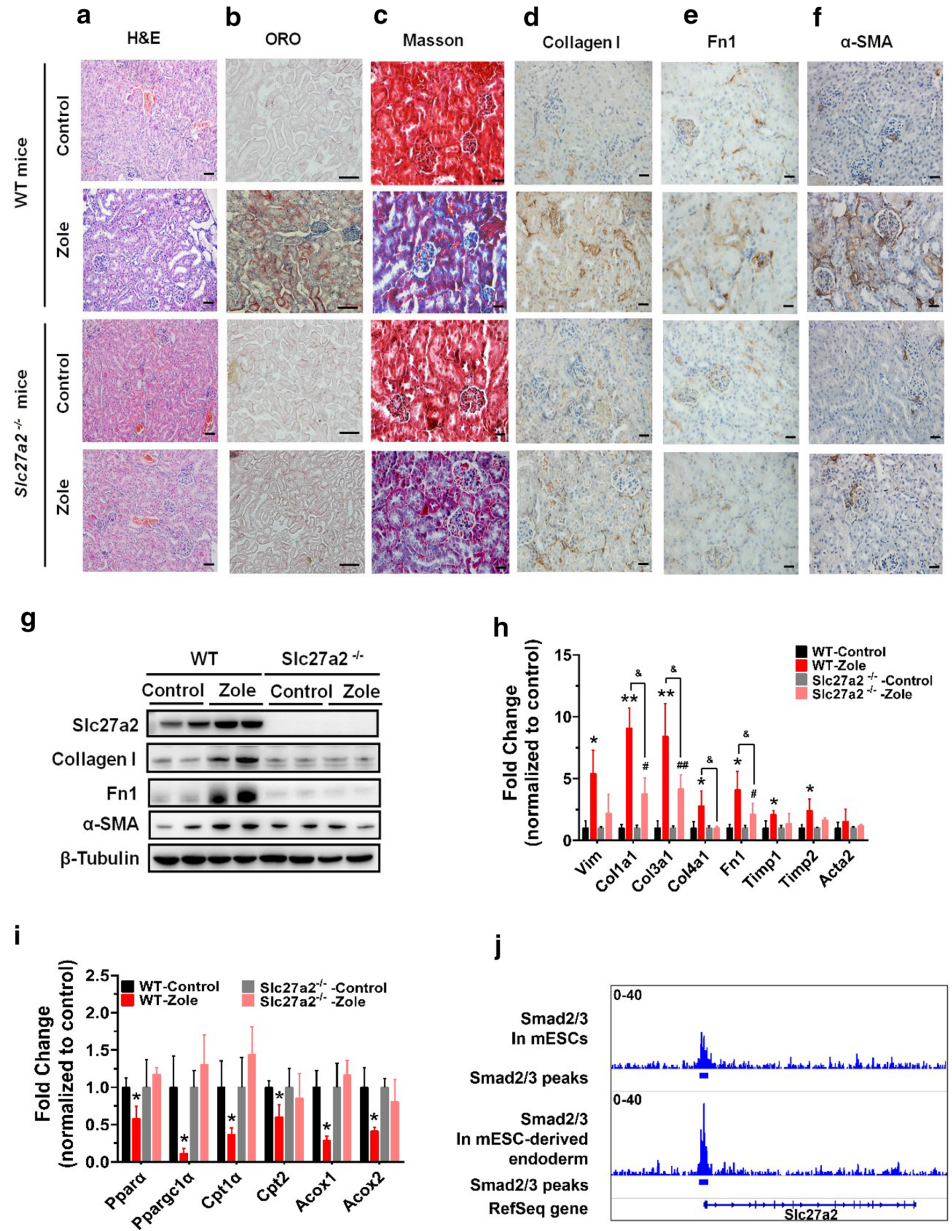
Effects of renal toxicity of zoledronate in *Slc27a2* ablated mice and its relative molecular pathways. **a**–**f** Representative photomicrographs of the H&E staining (*scale bar* 1 mm), ORO staining (*scale bar* 1 mm), Masson’s trichrome staining (*scale bar* 50 μm) and IHC images (*scale bar* 20 μm) of fibrosis markers of Fn1, collagen I and α-SMA from kidney sections in untreated WT and *Slc27a2* ^−*/*−^ mice as well as zoledronate-treated WT and *Slc27a2* ^−*/*−^ ones. **g** Immunoblotting of fibrosis markers of Fn1, collagen I and α-SMA in the mice above. **h** Relative mRNA levels of genes related to kidney fibrosis and injury from the samples above. **i** Relative transcript levels of FAO-related genes in untreated and zoledronate-treated WT and *Slc27a2* ^−*/*−^ mice. **j** Tracks of Smad2/3 ChIPseq in *Slc27a2* gene locus in mouse embryonic stem cells (mESCs) and mESC-derived endoderm cells. ^*#*^ *P* < *0.05,* ^*##*^ *P* < *0.01* (zoledronate-treated vs untreated *Slc27a2* ^−/−^ mice); ^*&*^ *P* < *0.05* (zoledronate-treated *Slc27a2* ^−/−^ mice vs treated WT ones)

Experiments in both HK-2 cells and mice demonstrated zoledronate treatment increases expression of FA transporter SLC27A2. SLC27A2 is unique amongst FA transport proteins as it functions as both a FA transporter and acyl-CoA synthetase that is important for FA synthesis and degradation, and expressed highly in kidney and liver (Anderson and Stahl 2013). We tested the hypothesis that reducing FA uptake mediated by Slc27a2 could improve zoledronate-induced renal toxicity. *Slc27a2* knockout (KO) mouse (*Slc27a2* ^−*/*−^) was generated using the CRISPR/Cas9 system (Fig. S5 online). *Slc27a2* ^−*/*−^ exposed to zoledronate did not display as severe signs of nephrotoxicity as wild type (WT) mice, as demonstrated by better kidney morphology (Fig. 5a) and no significant creatinine reduction (Fig. S6a online). Lipid accumulation, as indicated by ORO staining, was notably less in zoledronate-treated *Slc27a2* ^−*/*−^ mice as compared to zoledronate-treated WTs (Fig. 5b). Masson’s trichrome staining, collagen I, Fn1 and α-SMA IHC showed that kidney fibrosis severity was markedly reduced in zoledronate-treated *Slc27a2* ^−*/*−^ mice as compared with zoledronate-treated WT mice (Fig. 5c–f). To quantitatively measure the fibrosis damage, the positive areas of Masson’s staining were quantified and the fibrotic damage from zoledronate-treated Slc27a2 ablated mice was about 72.8% of the zoledronate-treated WT mice, indicating that Slc27a2 deficiency greatly reduced the zoledronate-induced nephrotoxicity (Fig. S6c). Consistent with our histological analysis, immunoblot and qPCR experiments for fibrosis markers including Fn1, collagen I and α-SMA were significantly lower or returned to basal levels in *Slc27a2*-deficient mice as compared to WT after zoledronate treatment (Fig. 5g). *Vim, Col4a1, Timp1* and *Timp2* were not significantly increased in zoledronate-treated *Slc27a2* ^−/−^ mice, while transcript levels of fibrotic factors *Col1a1, Col3a1* and *Fn1* were significantly decreased between zoledronate-treated *Slc27a2* ^−/−^ mice and zoledronate-treated WT mice (Fig. 5h). The pro-inflammatory cytokine markers, Il6 and Tnfα, had no significant alternation in zoledronate-treated *Slc27a2* ^−*/*−^ mouse kidneys as compared with the untreated ones except for a 2.1-fold increase of Il1b (Fig. S6b), suggesting that deficiency of *Slc27a2* in mouse kidney greatly protected the mice from zoledronate-induced injury. Furthermore, *Slc27a2* ablation prevented zoledronate-induced decrease in FAO regulators *Pparα* and *Ppargc1α* and their downstream genes (Fig. 5i).

We next checked if elevated TGFβ signaling following zoledronate treatment could underlie increased expression of Slc27a2. We identified a *SMAD3* peak in the promoter region of *Slc27a2* gene (Fig. 5j) (Yoon et al. 2015). Tracks of *Smad2/3* ChIP-seq are shown in *Slc27a2* gene locus in mouse embryonic stem cells (mESCs) and mESC-derived endoderm cells. *Slc27a2* proximate promoter region indicates significant *Smad2/3* occupancy both in mouse embryonic stem cells (mESCs) and mESC-derived endoderm cells. These results indicate that TGFβ1 may potentially regulate *Slc27a2* transcript levels directly through elevated TGFβ-SMAD2/3 signaling. Collectively, this is compelling data that elevated *Slc27a2* expression is a major contributor to lipid accumulation and kidney fibrosis following zoledronate treatment.

### PPAR agonist fenofibrate ameliorated zoledronate-associated renal toxicity

**Fig. 6:**
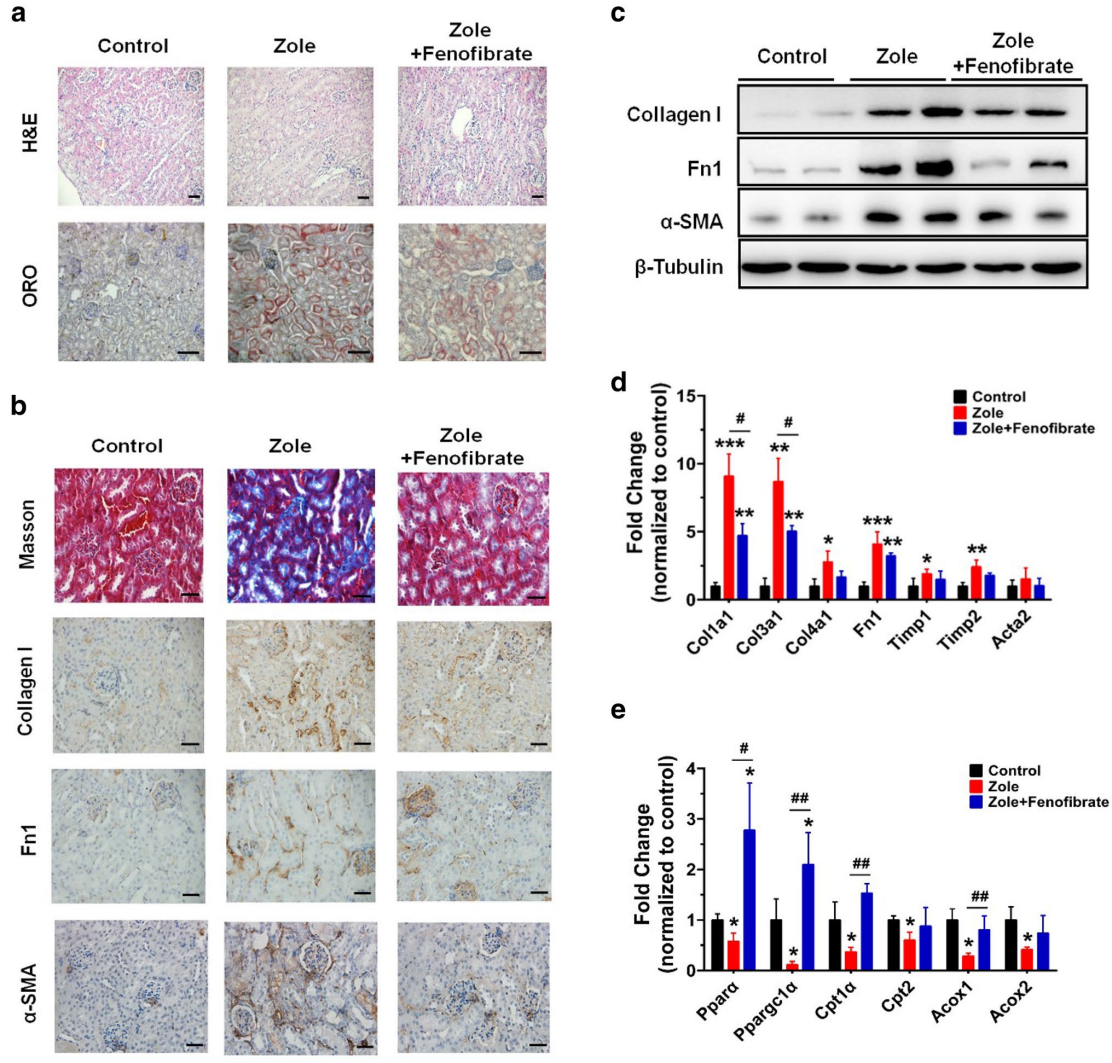
Effect of PPARA agonist fenobrinate on zoledronate-associated nephrotoxicity. **a** Representative images of H&E and ORO staining of kidney sections from control mice, zoledronate-treated mice and zoledronate-treated mice with fenofibrate (Zole + fenofibrate). (*scale bar* 1 mm). **b** Masson’s trichrome staining (scale bar: 50 μm) and fibrosis markers of Fn1, collagen I and α-SMA IHC (*scale bar* 20 μm) from kidney sections in control, zoledronate- and zoledronate + fenofibrate-treated mice. **c** Western blot analysis of kidney fibrosis markers in three groups of mice above. **d** Relative mRNA levels of genes related to kidney fibrosis from the samples above. **e** Relative transcript levels of FAO-related genes in the mice above. ^*#*^ *P* < *0.05,* ^*##*^ *P* < *0.01*, compared between zoledronate-treated or zoledronate + fenofibrate co-treated mice

Our studies in HK-2 cells and mice indicate that, following zoledronate treatment, elevated FA uptake via Slc27a2 may function in concert with impaired FAO to induce lipid accumulation. Thus we tested if pharmacological activation of the FAO regulator *PPARA* could ameliorate zoledronate-induced renal toxicity. Co-administration of fenofibrate, a *PPARA* agonist, with zoledronate decreased histological evidence of renal injury and lipid accumulation as compared with mice treated with zoledronate alone (Fig. 6a). Diminished fibrosis development and reduced fibrotic marker expressions were observed by Masson’s trichrome staining, collagen I, Fn1 and α-SMA IHC in fenofibrate and zoledronate co-administered group compared with those in the zoledronate alone group (Fig. 6b). Western blots confirmed co-treatment of fenofibrate and zoledronate resulted in lower expression of fibrotic markers (collagen I, Fn1 and α-SMA) in zoledronate and fenofibrate co-administration group as compared to zoledronate treatment alone (Fig. 6c). Fibrotic markers co-treatment treatment group were significantly lower (*Col1a1* and *Col3a1*) as compared to zoledronate however certain fibrotic genes (*Col4a1, Timp1* and *Timp2*) (Fig. 6d). FAO relevant genes (*Pparα, Ppargc1α, Cpt1α and Acox1*) were significantly increased after fenofibrate and zoledronate co-administration (Fig. 6e). These results indicate that restoring *PPARA* activity can partially alleviate the development of renal fibrosis by zoledronate.

## Discussion

In this study, we systematically investigated the molecular mechanism of zoledronate-induced kidney toxicity with human kidney tubular cell HK-2 and mouse models. Here we present activated TGFβ signaling triggering multiple dysregulated pathways in zoledronate-induced nephrotoxicity, including TGFβ/smad3 mediated profibrotic processes and abnormal FA metabolism. Our studies indicate that activated TGFβ signaling could be a major part of zoledronate-induced pathology in our cell and animal models. TGFβ is a well-established “master modulator” of profibrotic cytokine in fibrosis development of multiple tissues (Manickam et al. 2014; Zhan and Kanwar 2014). Our results from HK-2 cells and mouse kidney indicate zoledronate may elevate TGFβ signaling by increasing TGFβ1 protein expression. It may serve as a direct activator of TGFβ signaling in renal tubular cells and is a key mechanism contributing to zoledronate-induced kidney toxicity.

Along with increased TGFβ and renal fibrosis, we observed significant lipid accumulation in zoledronate-treated HK-2 cells and mice. Lipid accumulation in tubular epithelial cells has drawn increasing attention in acute, fibrotic and diabetic kidney diseases (Herman-Edelstein et al. 2014; Kang et al. 2015; Susztak et al. 2005). Excess deposition of triglyceride results in cellular lipotoxicity and promotes kidney fibrosis and injury (Kang et al. 2015). However, research into lipid metabolism in the kidney has been limited, and it is not clear if lipid accumulation is a cause or consequence of cellular toxicity. The lipid accumulation observed in both HK-2 and mouse kidney with zoledronate treatments may be downstream of zoledronate induced TGFβ signaling activation. TGFβ suppresses the PPARA’s expression in chronic kidney disease (CKD) patients with kidney fibrosis (Kang et al. 2015) and our in vitro and in vivo studies showed that PPARA or PPARGC1A was down-regulated by zoledronate treatments. Therefore, it is conceivable that down-regulation of PPARA or PPARGC1A and its target genes like CPTs and ACOXs following zoledronate treatment may inhibit the normal FAO in renal tubular cells and cause lipid accumulation. In support of this, we show that co-treatment with PPARA agonist fenofibrate partially ameliorated the effects of zoledronate-treatment on kidney fibrosis and lipid accumulation. Complementarily, a recent study showed that zoledronate decreased estrogen-related receptor α activity to down-regulate the activities of PPARA or PPARGC1A (Wei et al. 2016). Altogether, these evidences indicate that zoledronate has a negative regulation on FAO.

A parallelled mechanism for FA accumulation is increased expression of long chain FA transporter SLC27A2 following zoledronate-treatment. Importantly, our results indicate that SLC27A2 is the most highly expressed FA transporter in the kidney. Other studies point to CD36 as the main transporter and an important player in FA accumulation (Kang et al. 2015; Susztak et al. 2005). However, we found that CD36 gene expression was much lower than SLC27A2 and was not affected by zoledronate treatment. We provide compelling data using *Slc27a2* deficient mice that upregulation of the transporter following zoledronate treatment plays a major role in zoledronate-induced lipid accumulation and kidney fibrosis. Elevated SLC27A2 expression has been associated with proximal tubular toxicity in calcineurin inhibitor toxicity (Maluf et al. 2014), suggesting its role in drug mediated renal toxicity might be broader. Evidence has shown that SLC27A2 is a transcriptional target of Smad3 (Yoon et al. 2015), raising the possibility that elevated SLC27A2 results from zoledronate-induced increase in TGFβ activity. Our findings of impaired FA metabolism with zoledronate treatment indicate that drugs that block FA uptake or enhance FAO might be therapeutically beneficial for patients on zoledronate regiment or with kidney fibrosis.

**Fig. 7:**
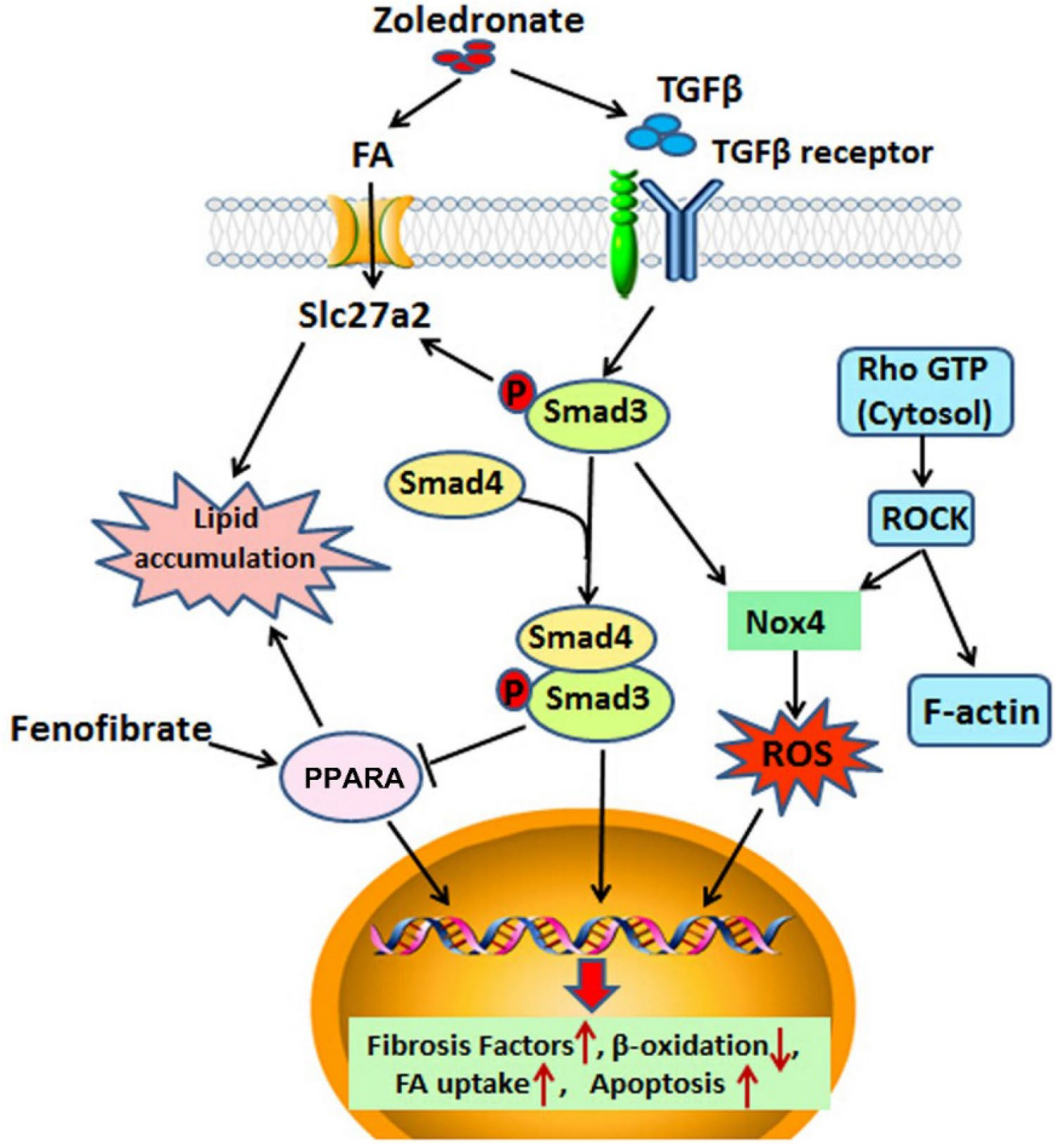
Summary of model of action of zoledronate-induced renal toxicity

Though our data support over-activation of TGFβ signaling as a major component of zoledronate-induced nephrotoxicity, our proteomic and metabolomic studies in zoledronate-treated HK-2 cells point to several potentially important pathways acting in parallel to and/or downstream of TGFβ signaling. The Rho family of GTPases, including CDC42 (2.7 ± 0.3 folds), RhoA (2.5 ± 0.3 folds) and Rac1 (1.8 ± 0.2 folds), were among the most elevated proteins identified in our proteomic data (Fig. S7a online). Furthermore, we identified increased Rho/Rac/CDC42 activity and actin stress fiber formation in zoledronate-treated HK-2 cells (Fig. S7b online). Zoledronate can inhibit protein prenelyation of small GTPases (Fig. S7c online) and this has been shown to cause accumulated cytosolic GTP-bound GTPase and sustained activation of downstream effector of RhoA, Rho kinase (Rock), NADPH oxidase 4 (Nox4)-derived ROS and Cofilin in HK-2 cells (Fig. S7d, e online), macrophages and osteoclasts (Dunford et al. 2006). Consequently, the ROS production was increased after zoledronate treatments (Fig. S8a online) consistent with the significant reduction in antioxidants like GSH from metabolomics assay (Fig. S8b online). The effect of zoledronate on GTPase prenylation and activity has been proposed as the mechanism of zoledronate associated renal toxicity (Markowitz et al. 2003; Munier et al. 2005; Ott 2012; Papapetrou 2009; Perazella and Markowitz 2008). Our work indicates zoledronate-induced FA accumulation and kidney fibrosis are the major factors in zoledronate induced nephrotoxicity however this does not rule out an important role for defective protein prenylation and altered GTPase activity in zoledronate kidney toxicity. TGFβ/Smad signaling can also increase GTPase activity and this may also contribute to elevated Rho/Rac/CDC42 activity in zoledronate-treated HK-2 cells (Manickam et al. 2014; Tsou et al. 2014; Zhan and Kanwar 2014). Collectively, these results suggested zoledronate activated TGFβ mediated multiple signaling pathways to induce nephrotoxicity (Fig. 7).

In summary, our study shows that zoledronate activates TGFβ signaling in the kidney to mediate several cellular events leading to renal toxicity, foremost of which are fibrosis development and dysregulation of FA metabolism. Restoring PPARA signaling to improve FAO or blocking FA transporter SLC27A2 with zoledronate treatments by pharmacological or genetic means helped protect mice from renal toxicity.

## **Electronic supplementary material**

**Below is the link to the electronic supplementary material.**

**Supplementary material 1 (DOCX 48 kb)**

**Supplementary material 2 (PPTX 4931 kb)**
