# Supplementary material for: Zoledronate dysregulates fatty acid metabolism in renal tubular epithelial cells to induce nephrotoxicity
Source: Arch Toxicol. 2017 Sep 4;92(1):469–85. doi: 10.1007/s00204-017-2048-0 (PMC5773652; doi:10.1007/s00204-017-2048-0)
Supplement: Supplementary file 1 — Supplementary material 1 (DOCX 48 kb) [file 204_2017_2048_MOESM1_ESM.docx]

**Table S1** **qPCR primers**

| **Gene name** | **Forward Primer** | **Reverse Primer** |
| --- | --- | --- |
| APAF1 | GTGGAATAACTTCGTATGTAAGGAC | CAACTGGCCTCTGTGGTACT |
| BAX | CATGGGCTGGACATTGGACT | GCAGCCCCCAACCACC |
| BCL2 | TGGGAGAACGGGGTACGATA | CATCTCCCGCATCCCACTC |
| KIM1 | TGGCAGATTCTGTAGCTGGTT | AGAGAACATGAGCCTCTATTCCA |
| TGFβ1 | CGACTCGCCAGAGTGGTTAT | TAGTGAACCCGTTGATGTCCA |
| VIM | CGAAAACACCCTGCAATCTT | AAGGTCAAGACGTGCCAGA |
| COL1A1 | GCGGACTTTGTTGCTGCTTGCAG | ATCTCCGGCTGGGCCCTTTCTT |
| COL2A1 | CGTTTCGCTGCGCTCC | CCAGCCTCCTGGACATCCT |
| COL3A1 | TCCTGGTCTGCAAGGAATGC | TTTCCCTGGGACACCATCAG |
| COL4A1 | CAAGGGCTCGCCGGGTTCTG | CCGGTGTCACCACGACTGCC |
| FN1 | AACAAACACTAATGTTAATTGCCCA | TCGGGAATCTTCTCTGTCAGC |
| TIMP1 | ATGCACAGTGTTTCCCTGTTTATC | CCTTTTCAGAGCCTTGGAGGA |
| TIMP2 | GTTTATCTACACGGCCCCCT | TCGGCCTTTCCTGCAATGAG |
| ACTA2 | GCCAAGCACTGTCAGGAATC | TTGTCACACACCAAGGCAGT |
| PPARA | GTGGACTCAACAGTTTGTGGC | CCGAGCTCCAAGCTACTCTT |
| PPARGC1A | CTCTCAGTAAGGGGCTGGTTG | AACCAGAGCAGCACACTCGAT |
| ACOX1 | CTTCAACCCGGAGCTGCTTA | ATGTTCTCGATCTCTCGGCG |
| ACOX2 | ACTGAAGCCACCTATGACGC | AGTCTCCAGGCCACCATTTG |
| CPT1A | GGAGACAGACACCATCCAGC | GCCAGACCTTGAAGTAGCGT |
| CPT2 | TGGTTGAGTGCTCCAAGTACC | GTCAAAGCCCTGGCCCATTG |
| SLC27A1 | CTCTCTCTGCTTCCCCAGGA | GCACAGAGAGACCGAAGAGG |
| SLC27A2 | ATCCCAGAGTCATGGAGGTCT | TGCTTTTGGAAGACCTGTGGT |
| SLC27A3 | GTAAGCCAGCAGTCCCCATT | ATTCTCCCCCTTCCACCTGA |
| SLC27A4 | CCACTCAGCAGGAAACATCG | GTGGCTGGTTCAGGAGGTAG |
| SLC27A5 | TTGTCGCAGGTGGACTTCTT | CACCTTACCCTCACAACCTGG |
| SLC27A6 | TTGAGTTGGGTGCCACTTGT | TTCCAATTGCCAAACGCACC |
| CD36 | TGATTGAAAAATCCTTCTTAGCCAT | ACAAGCTCTGGTTCTTATTCACAA |
| IL1B | TTCGACACATGGGATAACGAGG | TTTTTGCTGTGAGTCCCGGAG |
| IL6 | CCTGAACCTTCCAAAGATGGC | TTCACCAGGCAAGTCTCCTCA |
| TNFα | CCTCTCTCTAATCAGCCCTCTG | GAGGACCTGGGAGTAGATGAG |
| Vim | ACAAGGTTCGGGAAGAGGTT | CCGTGTAAGGGTCAAAGCAT |
| Col1a1 | GCTCTTTTTAGATACTGTGGTGAGGAA | GTTTCCACGTCTCACCATTG |
| Col2a1 | GGGAATGTCCTCTGCGATGAC | GAAGGGGATCTCGGGGTTG |
| Col3a1 | ACAGCTGGTGAACCTGGAAG | ACCAGGAGATCCATCTCGAC |
| Col4a1 | GACAGCCAGGTTTGACAGGT | GGCAGCTCTCTCCTTTCTGA |
| Fn1 | ACAAGGTTCGGGAAGAGGTT | CCGTGTAAGGGTCAAAGCAT |
| Timp1 | GCAACTCGGACCTGGTCATAA | CGGCCCGTGATGAGAAACT |
| Timp2 | TCAGAGCCAAAGCAGTGAGC | GCCGTGTAGATAAACTCGATGTC |
| Acta2 | CTGACAGAGGCACCACTGAA | AGAGGCATAGAGGGACAGCA |
| Apaf1 | GGGTGGGTCACCATCTATGG | TTCCGCAGCTAACACAGACTTG |
| Bax | TGAAGACAGGGGCCTTTTTG | AATTCGCCGGAGACACTCG |
| Bcl2 | TGGGATGCCTTTGTGGAACT | CAGCCAGGAGAAATCAAACAGA |
| Kim | GCTGCTACTGCTCCTTGTGA | GGAAGGCAACCACGCTTAGA |
| Ech1 | AAGATAAGGACGCCATGCTGAA | TCCAGGTGGCCATGTAGTCA |
| Mcad | GCTCGTGAGCACATTGAAAA | CATTGTCCAAAAGCCAAACC |
| Ehhadh | CCTGTTGCTCTTGACTTAATTACC | TTGATGGCTTCTTCAACTGG |
| Lcad | GCTGCCCTCCTCCCGATGTT | ATGTTTCTCTGCGATGTTGATG |
| Crat | CAGCCTCCATAGACTCGCTG | TCGGATGGCCCGGTCAG |
| Acaa1 | GCATCCCAGAGACTGTACCTTT | GTCTCTGGCCTTCTCGTTCT |
| Hsd17b4 | AGCTGTGAGGAAAATGGTGGC | TGATTCCGCTTTCTGACGATG |
| Hadhα | GGCAGTCTCAGTCGCTTCTC | GCACTCCTGATTTGGTCGTT |
| Slc25a20 | GTTCACCACAGGAATCATGAC | GTGAGCACAGTCCCTTTGTAG |
| Pparα | CAGCTGTTTTGGGGGCTG | TCAACTTGGCTCTCCTCTAAGTT |
| Ppargc1α | TCTCAGTAAGGGGCTGGTTG | AGCAGCACACTCTATGTCACTC |
| Cpt1α | TGAGCCTGGCCTCGCC | ATCTTGAGTGGTGACCGAGTCTG |
| Cpt2 | TGAGAACGGCATTGGGAAGG | ATCAAACCAGGGGCCTGAGA |
| Acox1 | ACCTTGCTGAATCAGGGCAC | AGATGAGTTCCGTGGCCCAT |
| Acox2 | CAGCAGGGCCTCATGCAATA | GGTACCAAGAACCTCTGTCCTG |
| Slc27a1 | CAAGCTCCAGCACAGGATG | GTCCCAGAAACCAAAGCAGC |
| Slc27a2 | TCCTCCAAGATGTGCGGTACT | TAGGTGAGCGTCTCGTCTCG |
| Slc27a3 | ACCTTCAGGTGGAAGGGAGA | GTCTCCAAGACCTCAGCCAC |
| Slc27a4 | ACTGTTCTCCAAGCTAGTGCT | GATGAAGACCCGGATGAAACG |
| Slc27a5 | GTAACGTCCCTGAGCAACCA | ACACATTTGCCCGAAGTCCA |
| Slc27a6 | TCCTCAACTCCAACCTTCGC | GCTTCCAAGCAAATCTCCGC |
| Cd36 | ATGGGCTGTGATCGGAACTG | GTCTTCCCAATAAGCATGTCTCC |
| Il1β | GCAACTGTTCCTGAACTCAACT | ATCTTTTGGGGTCCGTCAACT |
| Il6 | TAGTCCTTCCTACCCCAATTTCC | TTGGTCCTTAGCCACTCCTTC |
| Tnfα | CCCTCACACTCAGATCATCTTCT | GCTACGACGTGGGCTACAG |

**Table S2 Proteomics data**

| **Accession** | **Description** | | **Score** | **Coverage** | **Unique Peptides** | **Ratio (Zoledronate/**  **Control)** |
| --- | --- | --- | --- | --- | --- | --- |
| **Inflammation (TGF-beta Signaling, extracellular matrix, fibrosis et,al)** | | |  |  |  |  |
| B1AP15 | CD55 antigen, decay accelerating factor for complement (Cromer blood group), isoform CRA_d OS=Homo sapiens GN=CD55 PE=1 SV=1 - [B1AP15_HUMAN] | | 2.16 | 4.42 | 1 | 2.956 |
| P02771 | Alpha-fetoprotein OS=Homo sapiens GN=AFP PE=1 SV=1 - [FETA_HUMAN] | | 23.70 | 4.43 | 3 | 2.446 |
| P31431-2 | Isoform 2 of Syndecan-4 OS=Homo sapiens GN=SDC4 - [SDC4_HUMAN] | | 6.88 | 6.54 | 1 | 2.444 |
| Q5H9A7 | Metalloproteinase inhibitor 1 OS=Homo sapiens GN=TIMP1 PE=1 SV=1 - [Q5H9A7_HUMAN] | | 41.43 | 60.84 | 7 | 2.402 |
| Q9Y2G9-3 | Isoform 2 of Protein strawberry notch homolog 2 OS=Homo sapiens GN=SBNO2 - [SBNO2_HUMAN] | | 6.11 | 1.45 | 1 | 2.270 |
| P23142 | Fibulin-1 OS=Homo sapiens GN=FBLN1 PE=1 SV=4 - [FBLN1_HUMAN] | | 5.91 | 4.41 | 3 | 2.227 |
| P03973 | Antileukoproteinase OS=Homo sapiens GN=SLPI PE=1 SV=2 - [SLPI_HUMAN] | | 12.51 | 45.45 | 6 | 2.133 |
| P49747-2 | Isoform 2 of Cartilage oligomeric matrix protein OS=Homo sapiens GN=COMP - [COMP_HUMAN] | | 4.12 | 1.85 | 1 | 1.895 |
| A0A087WW43 | Inter-alpha-trypsin inhibitor heavy chain H3 OS=Homo sapiens GN=ITIH3 PE=4 SV=1 - [A0A087WW43_HUMAN] | | 4.45 | 4.18 | 1 | 1.803 |
| P02788-2 | Isoform DeltaLf of Lactotransferrin OS=Homo sapiens GN=LTF - [TRFL_HUMAN] | | 15.77 | 4.35 | 3 | 1.696 |
| E9PBF6 | Lamin-B1 OS=Homo sapiens GN=LMNB1 PE=1 SV=1 - [E9PBF6_HUMAN] | | 365.58 | 85.53 | 1 | 1.677 |
| D6RFL4 | Monocyte differentiation antigen CD14, urinary form (Fragment) OS=Homo sapiens GN=CD14 PE=4 SV=1 - [D6RFL4_HUMAN] | | 16.17 | 36.15 | 5 | 1.666 |
| P20908 | Collagen alpha-1(V) chain OS=Homo sapiens GN=COL5A1 PE=1 SV=3 - [CO5A1_HUMAN] | | 6.37 | 1.25 | 2 | 1.656 |
| Q8WUH2 | Transforming growth factor-beta receptor-associated protein 1 OS=Homo sapiens GN=TGFBRAP1 PE=1 SV=1 - [TGFA1_HUMAN] | | 2.08 | 2.09 | 1 | 1.545 |
| O43294-2 | Isoform 2 of Transforming growth factor beta-1-induced transcript 1 protein OS=Homo sapiens GN=TGFB1I1 - [TGFI1_HUMAN] | | 6.80 | 11.49 | 4 | 1.532 |
| P62877 | E3 ubiquitin-protein ligase RBX1 OS=Homo sapiens GN=RBX1 PE=1 SV=1 - [RBX1_HUMAN] | | 7.62 | 15.74 | 2 | 0.783 |
| Q8WUQ7 | Cactin OS=Homo sapiens GN=CACTIN PE=1 SV=3 - [CATIN_HUMAN] | | 9.43 | 4.75 | 2 | 0.745 |
| P63208 | S-phase kinase-associated protein 1 OS=Homo sapiens GN=SKP1 PE=1 SV=2 - [SKP1_HUMAN] | | 188.74 | 96.32 | 1 | 0.642 |
| Q96T58 | Msx2-interacting protein OS=Homo sapiens GN=SPEN PE=1 SV=1 - [MINT_HUMAN] | | 10.37 | 0.96 | 3 | 0.633 |
| K7ELY9 | Beclin-1 (Fragment) OS=Homo sapiens GN=BECN1 PE=4 SV=1 - [K7ELY9_HUMAN] fibrosis | | 12.68 | 16.16 | 4 | 0.590 |
| Q13330-3 | Isoform 3 of Metastasis-associated protein MTA1 OS=Homo sapiens GN=MTA1 - [MTA1_HUMAN] | | 65.35 | 34.38 | 2 | 0.564 |
| **Kidney injury marker** | |  |  |  |  |  |
| Q5H9A7 | Metalloproteinase inhibitor 1 OS=Homo sapiens GN=TIMP1 PE=1 SV=1 - [Q5H9A7_HUMAN] | | 41.43 | 60.84 | 7 | 2.402 |
| P16035 | Metalloproteinase inhibitor 2 OS=Homo sapiens GN=TIMP2 PE=1 SV=2 - [TIMP2_HUMAN] | | 3.83 | 9.09 | 1 | 2.336 |
| P01034 | Cystatin-C OS=Homo sapiens GN=CST3 PE=1 SV=1 - [CYTC_HUMAN] | | 9.20 | 25.34 | 2 | 2.253 |
| P02768 | Serum albumin OS=Homo sapiens GN=ALB PE=1 SV=2 - [ALBU_HUMAN] | | 480.24 | 13.30 | 12 | 1.575 |
| **Lipid & Fatty acid metabolism** | | |  |  |  |  |
| F5H001 | Oxidized low-density lipoprotein receptor 1, soluble form (Fragment) OS=Homo sapiens GN=OLR1 PE=4 SV=1 - [F5H001_HUMAN] | | 5.41 | 22.12 | 1 | 3.417 |
| G3V1R7 | Solute carrier family 27 (Fatty acid transporter), member 2, isoform CRA_b OS=Homo sapiens GN=SLC27A2 PE=1 SV=1 - [G3V1R7_HUMAN] | | 2.55 | 6.49 | 1 | 1.858 |
| E5RFU2 | Lipid-phosphate phosphatase OS=Homo sapiens GN=EPHX2 PE=1 SV=1 - [E5RFU2_HUMAN] | | 15.14 | 16.25 | 4 | 1.581 |
| Q9BX68 | Histidine triad nucleotide-binding protein 2, mitochondrial OS=Homo sapiens GN=HINT2 PE=1 SV=1 - [HINT2_HUMAN] | | 6.20 | 12.27 | 1 | 1.539 |
| H0Y9U7 | Long-chain-fatty-acid--CoA ligase 1 (Fragment) OS=Homo sapiens GN=ACSL1 PE=1 SV=1 - [H0Y9U7_HUMAN] | | 2.85 | 3.74 | 1 | 0.824 |
| O95573 | Long-chain-fatty-acid--CoA ligase 3 OS=Homo sapiens GN=ACSL3 PE=1 SV=3 - [ACSL3_HUMAN] | | 13.28 | 5.83 | 2 | 0.774 |
| Q15067 | Peroxisomal acyl-coenzyme A oxidase 1 OS=Homo sapiens GN=ACOX1 PE=1 SV=3 - [ACOX1_HUMAN] | | 8.63 | 7.12 | 4 | 0.767 |
| **TCA cycle & Oxidative phosphorylation** | | |  |  |  |  |
| P48735 | Isoform 2 of Isocitrate dehydrogenase [NADP], mitochondrial OS=Homo sapiens GN=IDH2 - [IDHP_HUMAN] | | 7.49193 | 9.25 | 1 | 1.853 |
| P11177 | Isoform 2 of Pyruvate dehydrogenase E1 component subunit beta, mitochondrial OS=Homo sapiens GN=PDHB - [ODPB_HUMAN] | | 14.7347 | 14.96 | 3 | 1.711 |
| P31930 | Cytochrome b-c1 complex subunit 1, mitochondrial OS=Homo sapiens GN=UQCRC1 PE=1 SV=3 - [QCR1_HUMAN] | | 7.63928 | 7.29 | 2 | 0.756 |
| P07919 | Cytochrome b-c1 complex subunit 6, mitochondrial OS=Homo sapiens GN=UQCRH PE=1 SV=2 - [QCR6_HUMAN] | | 21.4711 | 58.24 | 4 | 0.704 |
| **Ras signaling** |  | |  |  |  |  |
| P60953 | Cell division control protein 42 homolog OS=Homo sapiens GN=CDC42 PE=1 SV=2 - [CDC42_HUMAN] | | 101.957 | 48.69 | 10 | 2.660 |
| P61586 | Transforming protein RhoA OS=Homo sapiens GN=RHOA PE=1 SV=1 - [RHOA_HUMAN] | | 98.785 | 50.78 | 7 | 2.529 |
| P62745 | Rho-related GTP-binding protein RhoB OS=Homo sapiens GN=RHOB PE=1 SV=1 - [RHOB_HUMAN] | | 46.3731 | 54.59 | 7 | 2.304 |
| P63000 | Ras-related C3 botulinum toxin substrate 1 OS=Homo sapiens GN=RAC1 PE=1 SV=1 - [RAC1_HUMAN] | | 48.7543 | 39.58 | 10 | 1.846 |
| P84095 | Rho-related GTP-binding protein RhoG OS=Homo sapiens GN=RHOG PE=1 SV=1 - [RHOG_HUMAN] | | 14.0933 | 15.71 | 1 | 1.726 |
| P01112 | GTPase HRas OS=Homo sapiens GN=HRAS PE=1 SV=1 - [RASH_HUMAN] | | 22.4771 | 49.74 | 2 | 1.919 |
| P01116-2 | Isoform 2B of GTPase KRas OS=Homo sapiens GN=KRAS - [RASK_HUMAN] | | 26.0152 | 47.87 | 2 | 1.697 |
| E9PK85 | Ras-related protein R-Ras2 (Fragment) OS=Homo sapiens GN=RRAS2 PE=1 SV=1 - [E9PK85_HUMAN] | | 7.31192 | 19.88 | 2 | 1.508 |
| P11233 | Ras-related protein Ral-A OS=Homo sapiens GN=RALA PE=1 SV=1 - [RALA_HUMAN] | | 2.12296 | 5.83 | 1 | 1.500 |
| F5H157 | Ras-related protein Rab-35 (Fragment) OS=Homo sapiens GN=RAB35 PE=1 SV=1 - [F5H157_HUMAN] | | 22.2975 | 27.03 | 3 | 2.536 |
| Q9BXF6 | Rab11 family-interacting protein 5 OS=Homo sapiens GN=RAB11FIP5 PE=1 SV=1 - [RFIP5_HUMAN] | | 15.8194 | 6.58 | 2 | 2.023 |
| A8MXF6 | Rab-like protein 2B OS=Homo sapiens GN=RABL2B PE=4 SV=1 - [A8MXF6_HUMAN] | | 3.14545 | 10.91 | 1 | 1.888 |
| P61026 | Ras-related protein Rab-10 OS=Homo sapiens GN=RAB10 PE=1 SV=1 - [RAB10_HUMAN] | | 36.9573 | 31 | 4 | 1.715 |
| P51151 | Ras-related protein Rab-9A OS=Homo sapiens GN=RAB9A PE=1 SV=1 - [RAB9A_HUMAN] | | 17.4263 | 18.91 | 3 | 1.737 |
| P51149 | Ras-related protein Rab-7a OS=Homo sapiens GN=RAB7A PE=1 SV=1 - [RAB7A_HUMAN] | | 32.2466 | 49.76 | 8 | 1.663 |
| Q5SX86 | Rab GDP dissociation inhibitor beta (Fragment) OS=Homo sapiens GN=GDI2 PE=1 SV=1 - [Q5SX86_HUMAN] | | 82.002 | 80 | 1 | 1.600 |
| Q8WUD1 | Ras-related protein Rab-2B OS=Homo sapiens GN=RAB2B PE=1 SV=1 - [RAB2B_HUMAN] | | 20.1161 | 31.48 | 1 | 1.519 |
| P62834 | Ras-related protein Rap-1A OS=Homo sapiens GN=RAP1A PE=1 SV=1 - [RAP1A_HUMAN] | | 12.2366 | 20.65 | 3 | 2.185 |
| P50151 | Guanine nucleotide-binding protein G(I)/G(S)/G(O) subunit gamma-10 OS=Homo sapiens GN=GNG10 PE=1 SV=1 - [GBG10_HUMAN] | | 2.75284 | 19.12 | 1 | 1.946 |
| P62879 | Guanine nucleotide-binding protein G(I)/G(S)/G(T) subunit beta-2 OS=Homo sapiens GN=GNB2 PE=1 SV=3 - [GBB2_HUMAN] | | 18.266 | 18.82 | 3 | 1.796 |
| H0Y8W2 | Guanine nucleotide-binding protein subunit beta-2-like 1 (Fragment) OS=Homo sapiens GN=GNB2L1 PE=1 SV=1 - [H0Y8W2_HUMAN] | | 149.752 | 67.15 | 1 | 1.551 |
| B4DHR0 | Rab GTPase-binding effector protein 2 OS=Homo sapiens GN=RABEP2 PE=1 SV=1 - [B4DHR0_HUMAN] | | 5.34299 | 4.22 | 2 | 0.764 |
| P52565 | Rho GDP-dissociation inhibitor 1 OS=Homo sapiens GN=ARHGDIA PE=1 SV=3 - [GDIR1_HUMAN] | | 145.324 | 61.27 | 13 | 0.737 |
| F5H8L0 | Rab GTPase-activating protein 1-like OS=Homo sapiens GN=RABGAP1L PE=1 SV=1 - [F5H8L0_HUMAN] | | 16.704 | 12.99 | 2 | 0.733 |
| A5YM69 | Rho guanine nucleotide exchange factor 35 OS=Homo sapiens GN=ARHGEF35 PE=1 SV=1 - [ARG35_HUMAN] | | 17.0753 | 18.39 | 1 | 0.670 |
| H0YEI0 | Ras association domain-containing protein 7 (Fragment) OS=Homo sapiens GN=RASSF7 PE=4 SV=1 - [H0YEI0_HUMAN] | | 4.64522 | 8.76 | 1 | 0.627 |
| **Ca^2+^ related** |  | |  |  |  |  |
| P52823 | Stanniocalcin-1 OS=Homo sapiens GN=STC1 PE=1 SV=1 - [STC1_HUMAN] | | 14.5995 | 19.03 | 4 | 2.758 |
| P02774 | Vitamin D-binding protein OS=Homo sapiens GN=GC PE=1 SV=1 - [VTDB_HUMAN] | | 41.3972 | 13.5 | 7 | 2.457 |
| P04731 | Metallothionein-1A OS=Homo sapiens GN=MT1A PE=1 SV=2 - [MT1A_HUMAN] | | 28.1633 | 62.3 | 2 | 2.101 |
| A0A087X208 | Agrin OS=Homo sapiens GN=AGRN PE=4 SV=1 - [A0A087X208_HUMAN] | | 17.9963 | 4.09 | 4 | 1.541 |
| Q5T5Y3-2 | Isoform 2 of Calmodulin-regulated spectrin-associated protein 1 OS=Homo sapiens GN=CAMSAP1 - [CAMP1_HUMAN] | | 22.6552 | 3.93 | 3 | 0.693 |
| Q16566 | Calcium/calmodulin-dependent protein kinase type IV OS=Homo sapiens GN=CAMK4 PE=1 SV=1 - [KCC4_HUMAN] | | 8.47472 | 6.77 | 2 | 0.676 |
| Q14257 | Reticulocalbin-2 OS=Homo sapiens GN=RCN2 PE=1 SV=1 - [RCN2_HUMAN] | | 124.619 | 44.48 | 13 | 0.660 |
| P62158 | Calmodulin OS=Homo sapiens GN=CALM1 PE=1 SV=2 - [CALM_HUMAN] | | 51.412 | 71.81 | 6 | 0.657 |
| H0YL33 | Annexin (Fragment) OS=Homo sapiens GN=ANXA2 PE=1 SV=1 - [H0YL33_HUMAN] | | 204.82 | 94.37 | 1 | 0.651 |
| P25815 | Protein S100-P OS=Homo sapiens GN=S100P PE=1 SV=2 - [S100P_HUMAN] | | 107.639 | 70.53 | 8 | 0.615 |
| Q15293 | Reticulocalbin-1 OS=Homo sapiens GN=RCN1 PE=1 SV=1 - [RCN1_HUMAN] | | 46.144 | 21.75 | 6 | 0.575 |
| R4GN98 | Protein S100 (Fragment) OS=Homo sapiens GN=S100A6 PE=1 SV=1 - [R4GN98_HUMAN] | | 60.8039 | 49.41 | 12 | 0.566 |
| **Cell morphology & skeleton** | | |  |  |  |  |
| Q96QU8-2 | Isoform 2 of Exportin-6 OS=Homo sapiens GN=XPO6 - [XPO6_HUMAN] | | 3.85701 | 2.07 | 1 | 2.496 |
| Q9H4B7 | Tubulin beta-1 chain OS=Homo sapiens GN=TUBB1 PE=1 SV=1 - [TBB1_HUMAN] | | 115.206 | 13.97 | 1 | 1.552 |
| Q15149-3 | Isoform 3 of Plectin OS=Homo sapiens GN=PLEC - [PLEC_HUMAN] | | 4108.35 | 82.01 | 2 | 0.755 |
| P13693 | Translationally-controlled tumor protein OS=Homo sapiens GN=TPT1 PE=1 SV=1 - [TCTP_HUMAN] | | 232.589 | 47.67 | 13 | 0.742 |
| O43707 | Alpha-actinin-4 OS=Homo sapiens GN=ACTN4 PE=1 SV=2 - [ACTN4_HUMAN] | | 683.784 | 71.02 | 50 | 0.723 |
| Q13813 | Spectrin alpha chain, non-erythrocytic 1 OS=Homo sapiens GN=SPTAN1 PE=1 SV=3 - [SPTN1_HUMAN] | | 358.428 | 32.48 | 64 | 0.719 |
| Q13813 | Spectrin alpha chain, non-erythrocytic 1 OS=Homo sapiens GN=SPTAN1 PE=1 SV=3 - [SPTN1_HUMAN] | | 358.428 | 32.48 | 64 | 0.719 |
| P07951 | Tropomyosin beta chain OS=Homo sapiens GN=TPM2 PE=1 SV=1 - [TPM2_HUMAN] | | 508.1 | 71.13 | 3 | 0.680 |
| Q8WUP2-3 | Isoform 3 of Filamin-binding LIM protein 1 OS=Homo sapiens GN=FBLIM1 - [FBLI1_HUMAN] | | 10.3286 | 11.59 | 3 | 0.666 |
| P63313 | Thymosin beta-10 OS=Homo sapiens GN=TMSB10 PE=1 SV=2 - [TYB10_HUMAN] | | 29.285 | 61.36 | 4 | 0.664 |
| P62328 | Thymosin beta-4 OS=Homo sapiens GN=TMSB4X PE=1 SV=2 - [TYB4_HUMAN] | | 48.2538 | 61.36 | 6 | 0.660 |
| P12814-2 | Isoform 2 of Alpha-actinin-1 OS=Homo sapiens GN=ACTN1 - [ACTN1_HUMAN] | | 529.971 | 60.54 | 38 | 0.646 |
| **Cell traffic (endocytosis et al)** | | |  |  |  |  |
| F8VZV1 | Protein MON2 homolog OS=Homo sapiens GN=MON2 PE=1 SV=1 - [F8VZV1_HUMAN] | | 4.40733 | 2.43 | 1 | 2.175 |
| P02765 | Alpha-2-HS-glycoprotein OS=Homo sapiens GN=AHSG PE=1 SV=1 - [FETUA_HUMAN] | | 42.0334 | 7.08 | 5 | 2.165 |
| Q96RL7-4 | Isoform 4 of Vacuolar protein sorting-associated protein 13A OS=Homo sapiens GN=VPS13A - [VP13A_HUMAN] | | 2.50602 | 0.62 | 1 | 1.788 |
| J3QRU4 | Vesicle-associated membrane protein 2 OS=Homo sapiens GN=VAMP2 PE=4 SV=2 - [J3QRU4_HUMAN] | | 29.5559 | 46.02 | 1 | 1.631 |
| P17066 | Heat shock 70 kDa protein 6 OS=Homo sapiens GN=HSPA6 PE=1 SV=2 - [HSP76_HUMAN] | | 190.505 | 22.86 | 2 | 1.541 |
| I3L3E4 | Charged multivesicular body protein 6 (Fragment) OS=Homo sapiens GN=CHMP6 PE=1 SV=1 - [I3L3E4_HUMAN] | | 1.88944 | 12.39 | 1 | 1.522 |
| P54652 | Heat shock-related 70 kDa protein 2 OS=Homo sapiens GN=HSPA2 PE=1 SV=1 - [HSP72_HUMAN] | | 389.232 | 24.1 | 2 | 1.500 |
| Q5VIR6-4 | Isoform 4 of Vacuolar protein sorting-associated protein 53 homolog OS=Homo sapiens GN=VPS53 - [VPS53_HUMAN] | | 15.9098 | 4.33 | 2 | 0.745 |
| P20645 | Cation-dependent mannose-6-phosphate receptor OS=Homo sapiens GN=M6PR PE=1 SV=1 - [MPRD_HUMAN] | | 10.2372 | 12.64 | 2 | 0.741 |
| Q9H1H9-3 | Isoform 3 of Kinesin-like protein KIF13A OS=Homo sapiens GN=KIF13A - [KI13A_HUMAN] | | 17.1169 | 3.49 | 2 | 0.730 |
| Q8N0X7 | Spartin OS=Homo sapiens GN=SPG20 PE=1 SV=1 - [SPG20_HUMAN] | | 43.3224 | 15.77 | 9 | 0.683 |
| **Cell cycle** |  | |  |  |  |  |
| P60953 | Cell division control protein 42 homolog OS=Homo sapiens GN=CDC42 PE=1 SV=2 - [CDC42_HUMAN] | | 101.957 | 48.69 | 10 | 2.660 |
| Q96FF9 | Sororin OS=Homo sapiens GN=CDCA5 PE=1 SV=1 - [CDCA5_HUMAN] | | 8.87586 | 15.87 | 3 | 1.754 |
| P49736 | DNA replication licensing factor MCM2 OS=Homo sapiens GN=MCM2 PE=1 SV=4 - [MCM2_HUMAN] | | 244.294 | 38.94 | 31 | 0.785 |
| E7EVZ2 | 14-3-3 protein zeta/delta (Fragment) OS=Homo sapiens GN=YWHAZ PE=1 SV=1 - [E7EVZ2_HUMAN] | | 177.784 | 82.65 | 2 | 0.745 |
| Q13416 | Origin recognition complex subunit 2 OS=Homo sapiens GN=ORC2 PE=1 SV=2 - [ORC2_HUMAN] | | 16.3955 | 15.08 | 5 | 0.742 |
| Q8N3U4 | Cohesin subunit SA-2 OS=Homo sapiens GN=STAG2 PE=1 SV=3 - [STAG2_HUMAN] | | 71.9866 | 15.03 | 12 | 0.707 |
| Q8NHZ8 | Anaphase-promoting complex subunit CDC26 OS=Homo sapiens GN=CDC26 PE=1 SV=1 - [CDC26_HUMAN] | | 19.0616 | 71.76 | 6 | 0.680 |
| P63208 | S-phase kinase-associated protein 1 OS=Homo sapiens GN=SKP1 PE=1 SV=2 - [SKP1_HUMAN] | | 188.742 | 96.32 | 1 | 0.642 |
| **Autophagy & phagocytosis & Lysosome** | | |  |  |  |  |
| Q2TAZ0-4 | Isoform 3 of Autophagy-related protein 2 homolog A OS=Homo sapiens GN=ATG2A - [ATG2A_HUMAN] | | 3.93128 | 7.55 | 1 | 1.799 |
| Q9UMX0 | Ubiquilin-1 OS=Homo sapiens GN=UBQLN1 PE=1 SV=2 - [UBQL1_HUMAN] | | 84.7873 | 32.09 | 5 | 1.537 |
| P06280 | Alpha-galactosidase A OS=Homo sapiens GN=GLA PE=1 SV=1 - [AGAL_HUMAN] | | 21.4529 | 12.59 | 4 | 0.789 |
| P11279 | Lysosome-associated membrane glycoprotein 1 OS=Homo sapiens GN=LAMP1 PE=1 SV=3 - [LAMP1_HUMAN] | | 21.1685 | 10.07 | 4 | 0.758 |
| P20645 | Cation-dependent mannose-6-phosphate receptor OS=Homo sapiens GN=M6PR PE=1 SV=1 - [MPRD_HUMAN] | | 10.2372 | 12.64 | 2 | 0.741 |
| Q14108 | Lysosome membrane protein 2 OS=Homo sapiens GN=SCARB2 PE=1 SV=2 - [SCRB2_HUMAN] | | 12.1202 | 8.37 | 4 | 0.728 |
| Q8N122-3 | Isoform 3 of Regulatory-associated protein of mTOR OS=Homo sapiens GN=RPTOR - [RPTOR_HUMAN] | | 6.72456 | 2.04 | 2 | 0.713 |

**Table S3 Metabolomics data**

| **Compound Name** | **Ratio (Zoledronate/**  **Control)** | **p-Value** |
| --- | --- | --- |
| Thymidine 5'-triphosphate | 8.83 | 1.23E-02 |
| Phytosphingosine | 5.93 | 5.88E-06 |
| Guanosine diphosphate | 4.71 | 2.48E-02 |
| Sphinganine | 4.68 | 4.34E-02 |
| Hexanoylcarnitine | 3.60 | 1.80E-03 |
| Oleic Acid | 3.56 | 4.26E-03 |
| Eicosanoyl-EA | 3.51 | 1.31E-03 |
| Histamine | 3.39 | 8.50E-04 |
| Uracil | 3.34 | 3.08E-04 |
| Adenine | 3.28 | 5.62E-03 |
| Isoleucine/Leucine | 3.13 | 4.38E-04 |
| N-arachidonoyl taurine | 3.05 | 7.91E-04 |
| L-Palmitoylcarnitine | 2.63 | 8.73E-03 |
| Urocanic acid | 2.53 | 5.34E-03 |
| Arachidonic Acid | 2.36 | 4.07E-02 |
| LysoPC(20:1) | 2.24 | 4.55E-02 |
| N-oleoyl taurine | 2.21 | 1.56E-03 |
| Flavin Mononucleotide | 2.15 | 1.63E-02 |
| Thiamine | 2.08 | 2.65E-02 |
| Oxoglutaric acid | 1.94 | 3.26E-02 |
| Uridine diphosphate glucuronic acid | 1.93 | 9.54E-03 |
| CDP | 1.92 | 3.32E-02 |
| N-Palmitoyl taurine | 1.89 | 2.07E-02 |
| Glucosamine/D-Galactosamine | 1.82 | 3.45E-02 |
| N-Acetyl-alpha-D-glucosamine 1-phosphate | 1.79 | 3.28E-02 |
| Palmitic acid | 1.77 | 1.67E-02 |
| Stearic acid | 1.73 | 2.22E-02 |
| Gluconic acid | 1.60 | 1.95E-02 |
| Uridine 5'-monophosphate | 1.47 | 1.96E-02 |
| FAD | 1.39 | 4.94E-02 |
| Myristoylcarnitine | 1.33 | 1.67E-02 |
| Glucosamine 6-phosphate | 0.84 | 1.82E-02 |
| Butyrylcarnitine | 0.82 | 3.66E-02 |
| Cytidine monophosphate | 0.70 | 2.95E-04 |
| Cytidine monophosphate | 0.70 | 2.95E-04 |
| Carnosine | 0.66 | 4.64E-02 |
| Uridine 5'-diphosphate | 0.65 | 9.68E-04 |
| ADP-glucose | 0.64 | 2.15E-02 |
| GDP-L-fucose | 0.64 | 2.15E-02 |
| Cytidine diphosphate choline | 0.63 | 3.28E-03 |
| Citicoline | 0.62 | 8.84E-03 |
| D-Aspartic acid | 0.61 | 2.25E-02 |
| Sphingosine | 0.61 | 4.92E-03 |
| L-Alpha-aminobutyric acid | 0.59 | 2.28E-02 |
| methacholine | 0.57 | 1.62E-05 |
| L-Alanine | 0.55 | 4.81E-02 |
| 4-Trimethylammoniobutanoic acid/Acetylcholine | 0.53 | 1.52E-03 |
| S-(1,2-Dicarboxyethyl) Glutathione | 0.53 | 1.81E-03 |
| Dimethylglycine | 0.51 | 3.42E-03 |
| N-Acetylaspartylglutamic acid | 0.46 | 1.65E-03 |
| Phosphoenolpyruvic acid | 0.46 | 1.50E-02 |
| Inosine | 0.45 | 2.84E-02 |
| Hypotaurine | 0.44 | 1.13E-02 |
| CDP-ethanolamine | 0.43 | 2.82E-03 |
| L-Acetylcarnitine | 0.41 | 3.92E-04 |
| L-Homocysteic acid | 0.41 | 1.99E-03 |
| L-a-Lysophosphatidylserine | 0.41 | 3.64E-02 |
| Fructose 1,6-bisphosphate | 0.40 | 3.53E-02 |
| 5-Aminoimidazole ribonucleotide | 0.40 | 8.97E-04 |
| Creatine | 0.39 | 3.28E-04 |
| L-Valine/5-Aminopentanoic acid | 0.39 | 2.02E-02 |
| Betaine | 0.39 | 9.23E-03 |
| Oxidized glutathione | 0.38 | 2.07E-02 |
| Uridine diphosphate-N-acetylglucosamine | 0.35 | 1.50E-05 |
| Carnitine | 0.34 | 2.93E-03 |
| Thymidine | 0.34 | 2.14E-03 |
| ADP | 0.33 | 3.16E-03 |
| Picolinic acid | 0.32 | 3.45E-05 |
| Flavone/L-Cystathionine | 0.30 | 2.17E-02 |
| L-Proline | 0.29 | 1.08E-02 |
| Glycerophosphocholine | 0.26 | 2.37E-03 |
| Cytosine | 0.25 | 2.61E-04 |
| Glutathione | 0.25 | 8.83E-03 |
| O-Phosphorylethanolamine | 0.24 | 1.66E-03 |
| N-Acetyl-L-aspartic acid | 0.23 | 1.02E-03 |
| N-Acetyl-L-glutamic acid | 0.23 | 2.57E-04 |
| Uridine monophosphate (UMP) | 0.21 | 1.13E-03 |
| Uridine diphosphate (UDP) | 0.20 | 2.33E-03 |
| Taurine | 0.19 | 4.99E-03 |
| Fumaric acid/ Maleic acid | 0.16 | 4.90E-05 |
| NADP+ | 0.16 | 1.21E-03 |
| Guanosine 5-diphosphate (GDP) | 0.16 | 2.00E-04 |
| Citric acid/Isocitrate | 0.15 | 4.63E-02 |
| Phosphocreatine | 0.15 | 1.01E-03 |
| NAD+ | 0.15 | 1.09E-03 |
| Inosine 5'-monophosphate (IMP) | 0.12 | 2.49E-03 |
| N-Acetyl-L-alanine | 0.08 | 4.75E-07 |
| L-Tyrosine | 0.07 | 2.64E-02 |
| N-acetylaspartate | 0.06 | 9.50E-07 |
| S-Adenosylmethionine | 0.05 | 1.24E-03 |
| Propionylcarnitine | 0.04 | 4.89E-03 |
