# Supplementary material for: Zoledronate dysregulates fatty acid metabolism in renal tubular epithelial cells to induce nephrotoxicity
Source: Arch Toxicol. 2017 Sep 4;92(1):469–85. doi: 10.1007/s00204-017-2048-0 (PMC5773652; doi:10.1007/s00204-017-2048-0)
Supplement: Supplementary file 2 — Supplementary material 2 (PPTX 4931 kb) [file 204_2017_2048_MOESM2_ESM.pptx]

## Slide 1
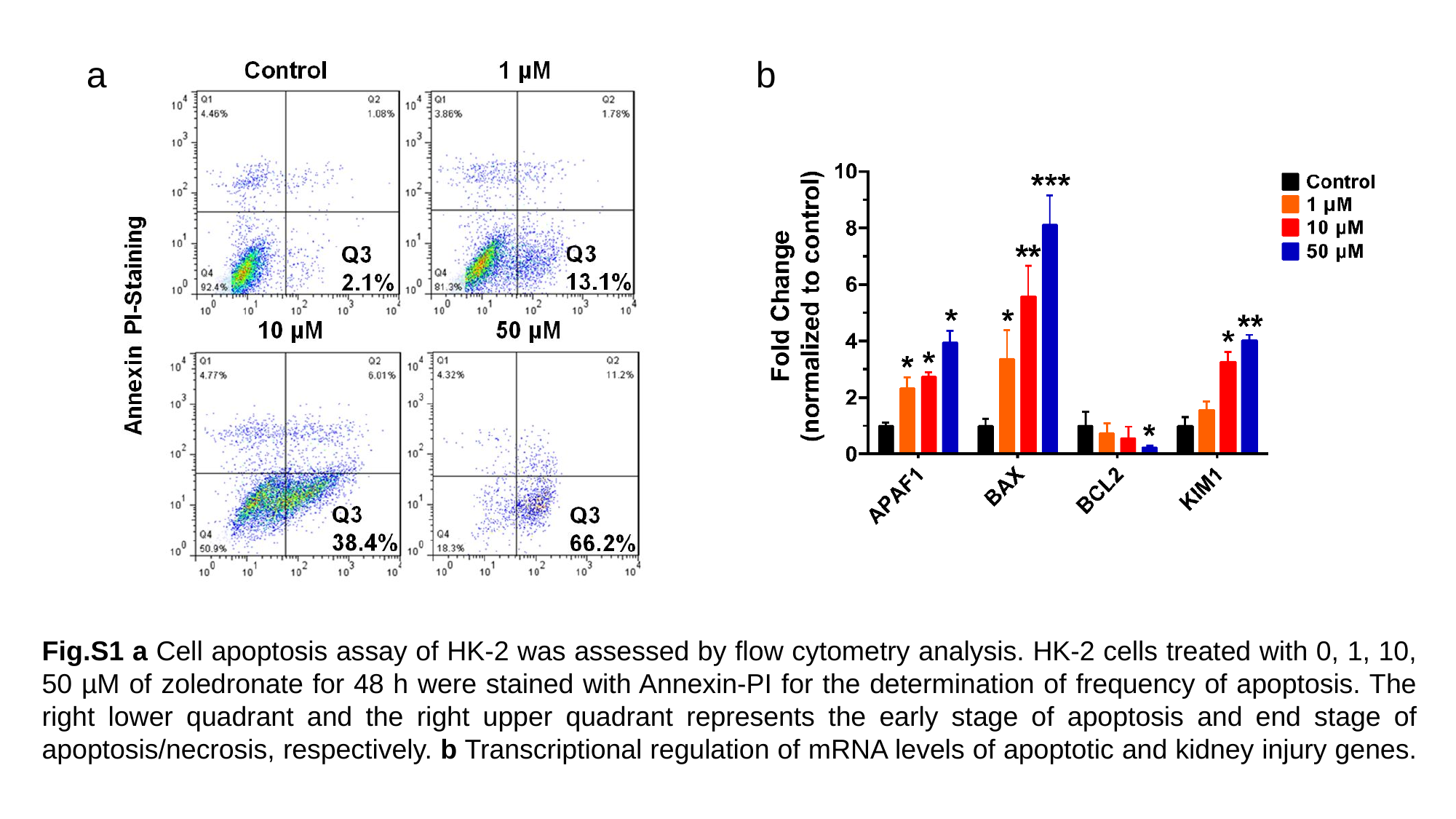

a b
Fig.S1 a Cell apoptosis assay of HK-2 was assessed by ﬂow cytometry analysis. HK-2 cells treated with 0, 1, 10, 50 µM of zoledronate for 48 h were stained with Annexin-PI for the determination of frequency of apoptosis. The right lower quadrant and the right upper quadrant represents the early stage of apoptosis and end stage of apoptosis/necrosis, respectively. b Transcriptional regulation of mRNA levels of apoptotic and kidney injury genes.

## Slide 2
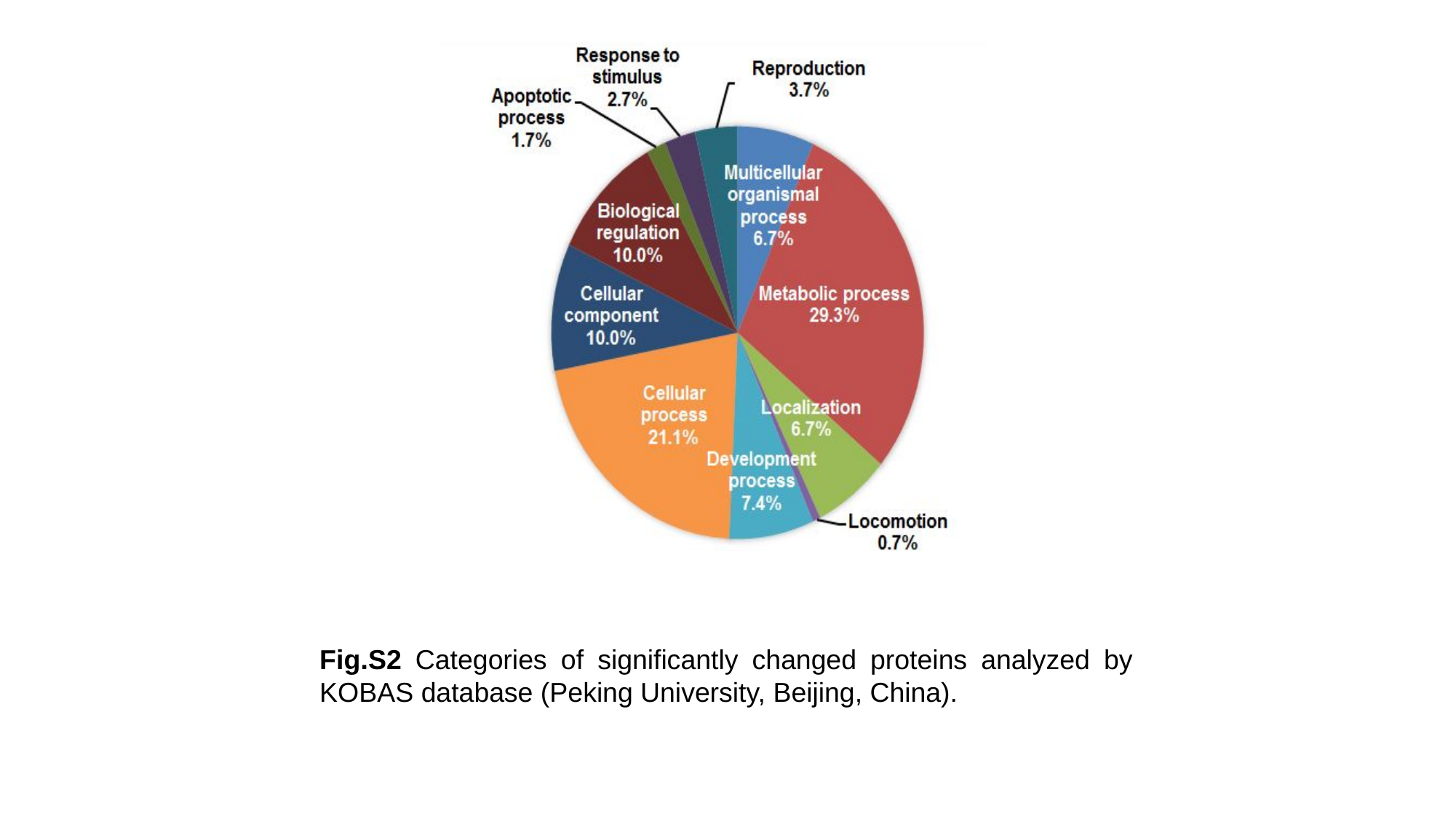

Fig.S2 Categories of significantly changed proteins analyzed by KOBAS database (Peking University, Beijing, China).

## Slide 3
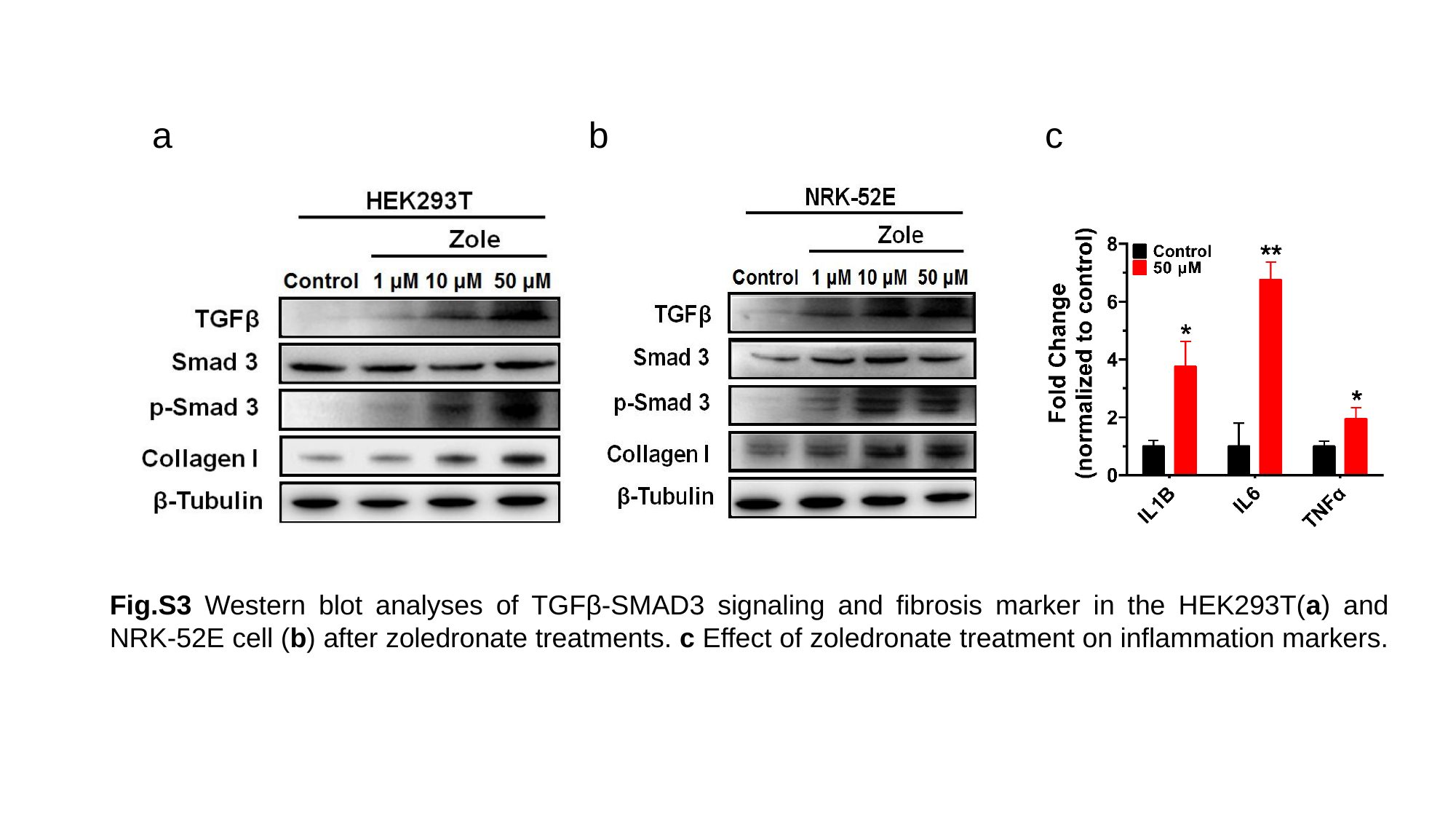

a b c
Fig.S3 Western blot analyses of TGFβ-SMAD3 signaling and fibrosis marker in the HEK293T(a) and NRK-52E cell (b) after zoledronate treatments. c Effect of zoledronate treatment on inflammation markers.

## Slide 4
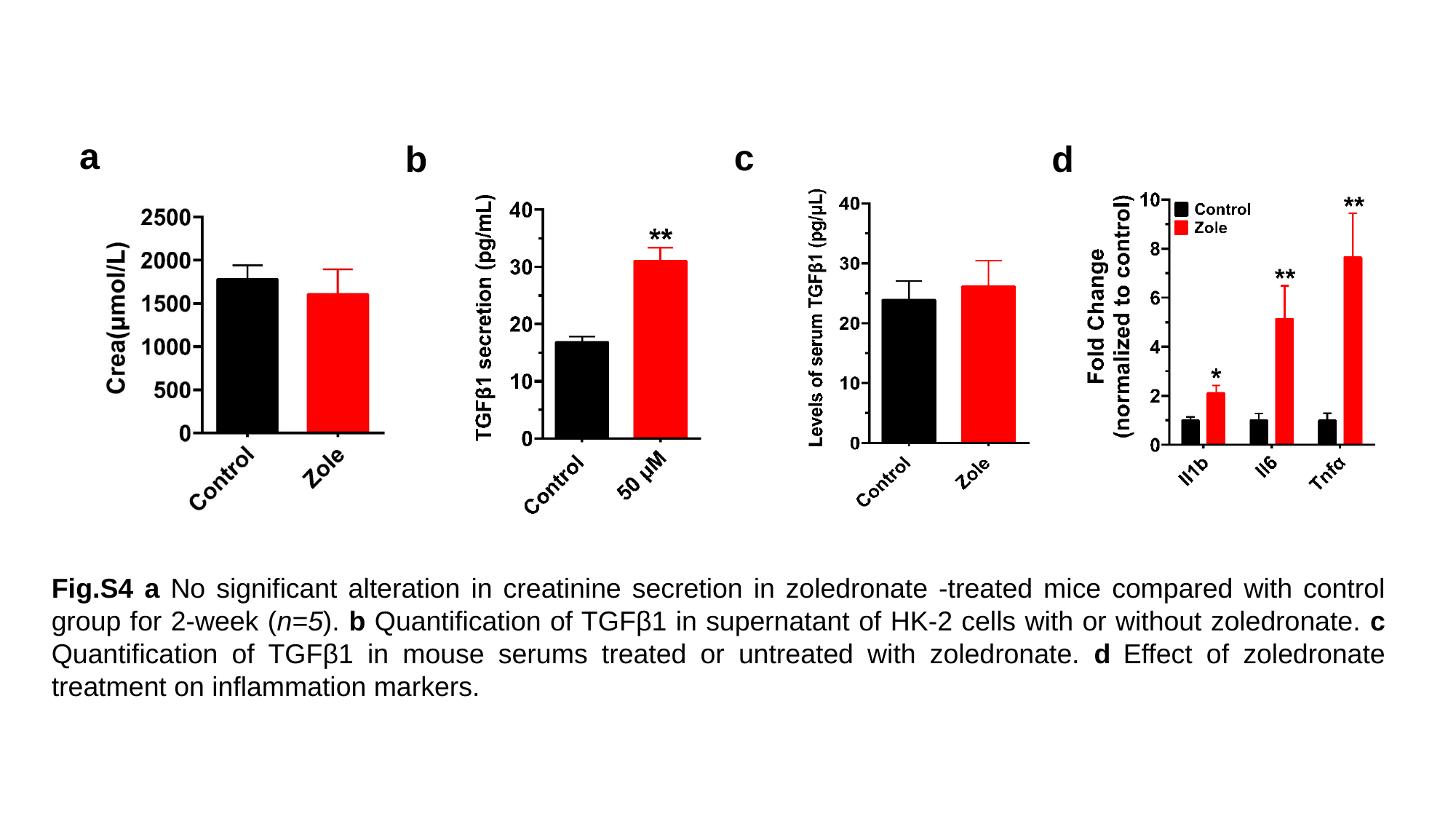

a
c
d
b
Fig.S4 a No significant alteration in creatinine secretion in zoledronate -treated mice compared with control group for 2-week (n=5). b Quantification of TGFβ1 in supernatant of HK-2 cells with or without zoledronate. c Quantification of TGFβ1 in mouse serums treated or untreated with zoledronate. d Effect of zoledronate treatment on inflammation markers.

## Slide 5
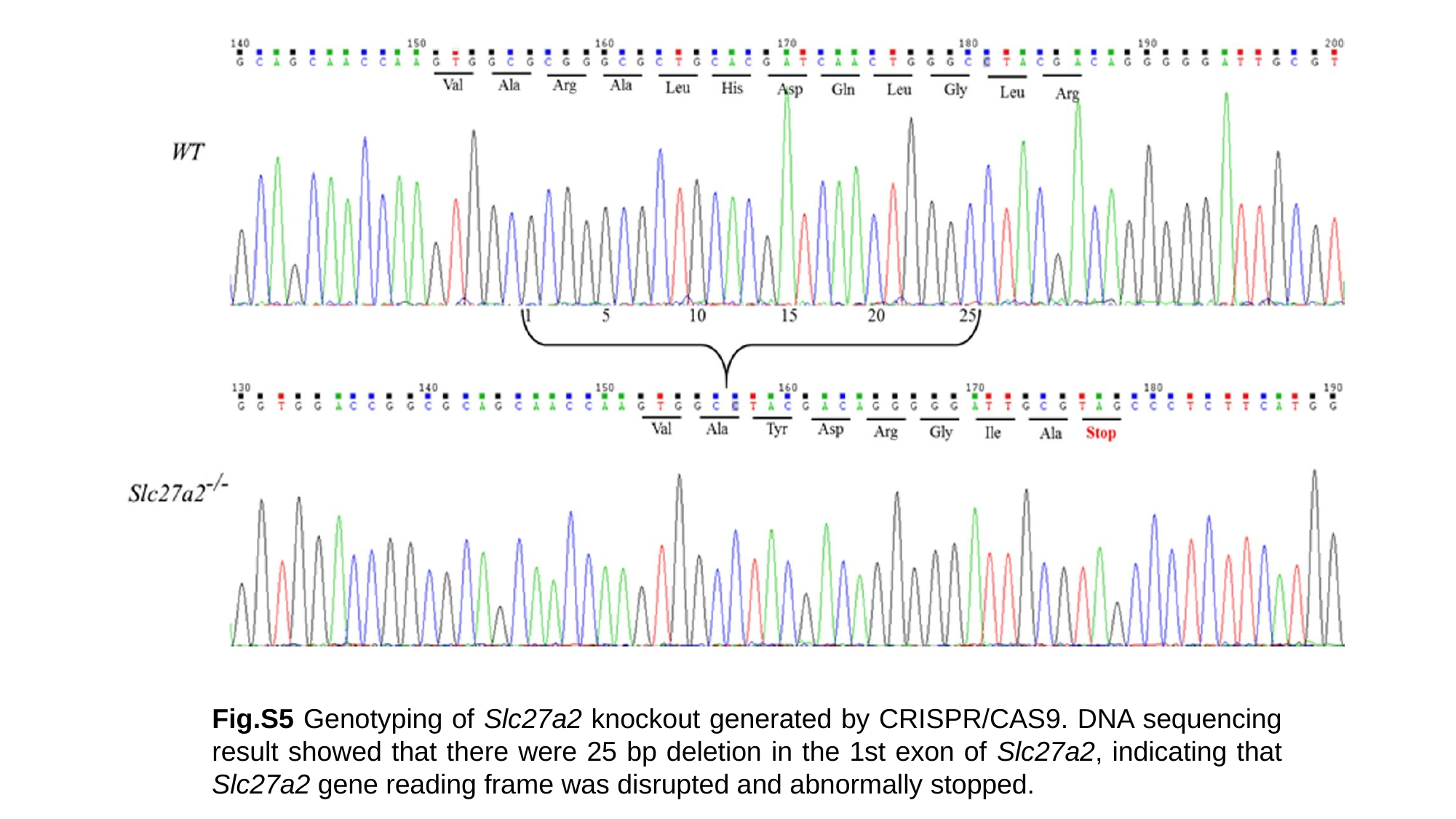

Fig.S5 Genotyping of Slc27a2 knockout generated by CRISPR/CAS9. DNA sequencing result showed that there were 25 bp deletion in the 1st exon of Slc27a2, indicating that Slc27a2 gene reading frame was disrupted and abnormally stopped.

## Slide 6
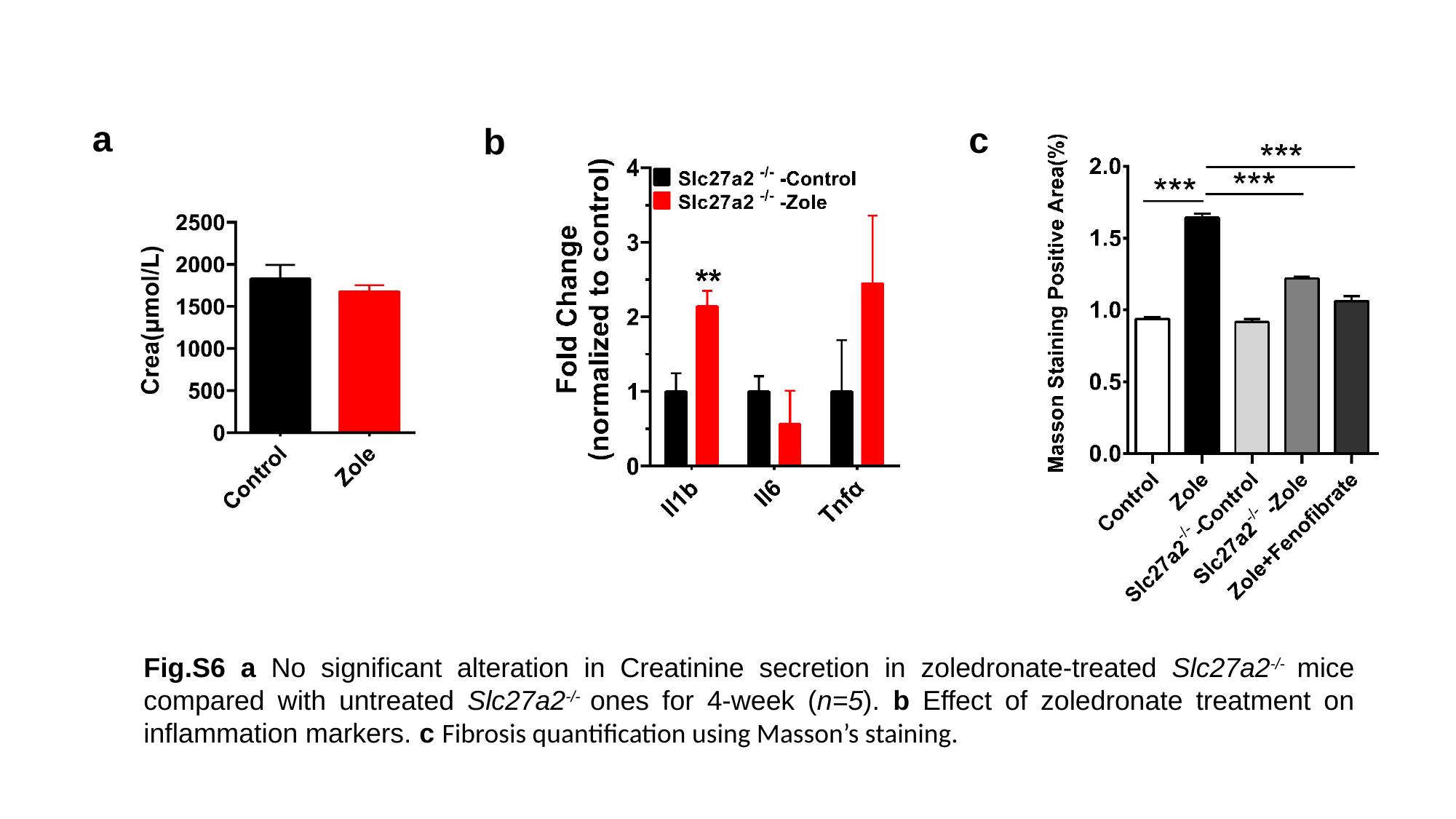

a
c
b
Fig.S6 a No significant alteration in Creatinine secretion in zoledronate-treated Slc27a2-/- mice compared with untreated Slc27a2-/- ones for 4-week (n=5). b Effect of zoledronate treatment on inflammation markers. c Fibrosis quantification using Masson’s staining.

## Slide 7
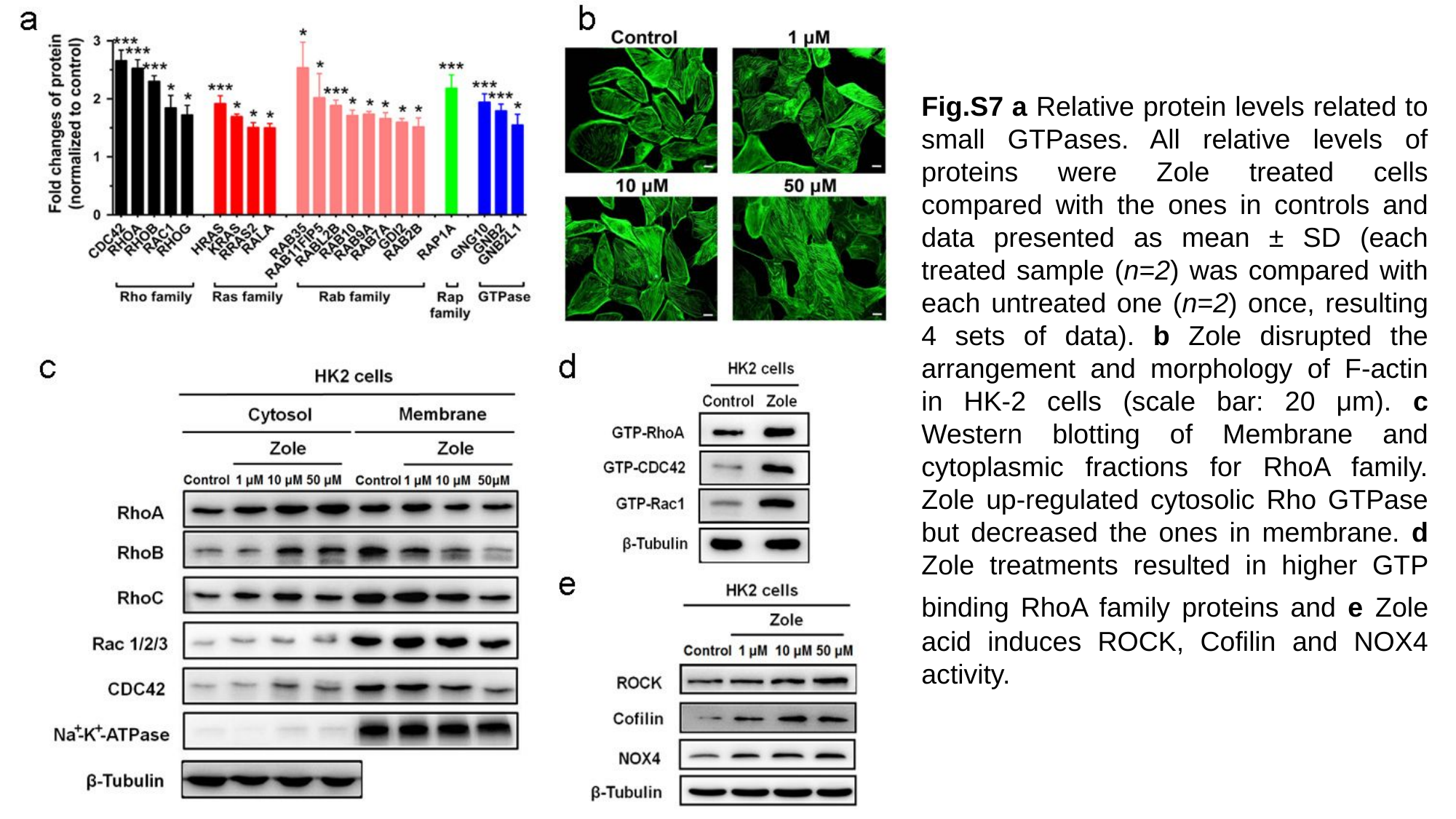

Fig.S7 a Relative protein levels related to small GTPases. All relative levels of proteins were Zole treated cells compared with the ones in controls and data presented as mean ± SD (each treated sample (n=2) was compared with each untreated one (n=2) once, resulting 4 sets of data). b Zole disrupted the arrangement and morphology of F-actin in HK-2 cells (scale bar: 20 μm). c Western blotting of Membrane and cytoplasmic fractions for RhoA family. Zole up-regulated cytosolic Rho GTPase but decreased the ones in membrane. d Zole treatments resulted in higher GTP binding RhoA family proteins and e Zole acid induces ROCK, Cofilin and NOX4 activity.

## Slide 8
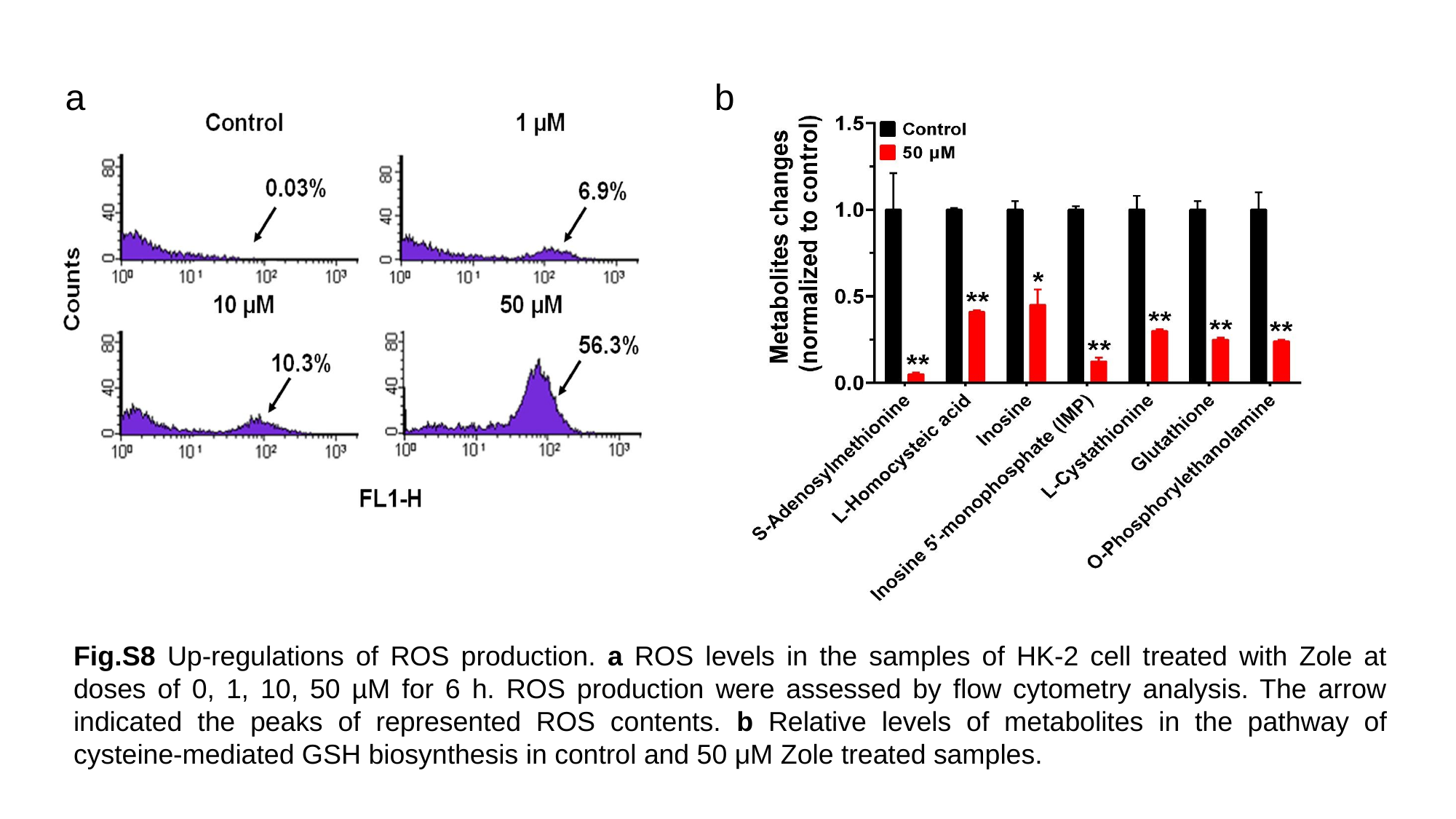

a b
Fig.S8 Up-regulations of ROS production. a ROS levels in the samples of HK-2 cell treated with Zole at doses of 0, 1, 10, 50 µM for 6 h. ROS production were assessed by ﬂow cytometry analysis. The arrow indicated the peaks of represented ROS contents. b Relative levels of metabolites in the pathway of cysteine-mediated GSH biosynthesis in control and 50 μM Zole treated samples.
